# Oxidative Stress-Related Metabolomic Alterations in Pregnancy: Evidence from Exposure to Air Pollution, Metals/Metalloid, and Tobacco Smoke

**DOI:** 10.3390/antiox14121442

**Published:** 2025-11-30

**Authors:** Alica Pizent

**Affiliations:** Division of Occupational and Environmental Health, Institute for Medical Research and Occupational Health, Ksaverska Cesta 2, HR-10000 Zagreb, Croatia; apizent@imi.hr

**Keywords:** environmental pollutants, metabolomics, amino acid metabolism, carbohydrate metabolism, lipid metabolism, oxidative stress

## Abstract

Developmental programming, shaped by environmental and lifestyle stressors during prenatal life, is increasingly recognized as a major contributor to non-communicable diseases (NCDs) later in life. Oxidative stress, one of key mechanisms linking these stressors to fetal metabolomic reprogramming and disease pathogenesis, leaves measurable metabolomic signatures that reflect disrupted redox balance. Alterations in glucose, lipid, and amino acid metabolism and antioxidant response could reveal the main pathways driving NCD development. This review summarizes epidemiological studies that have investigated biochemical responses of the prenatal exposure to metals, air pollution, and tobacco smoke and e-cigarette vapor in maternal–placental–fetal compartments using a metabolomic approach. Summarized studies indicate that maternal exposure to metals primarily disrupts amino acid pathways related to one-carbon metabolism, glutathione synthesis, and oxidative stress defense, while air pollution, particularly fine particulate matter, mainly affects lipid oxidation, fatty acid β-oxidation, and amino acid and carbohydrate metabolism. Tobacco smoke and e-cigarette vapor induce widespread disturbances involving reduced citric acid cycle intermediates, altered acylcarnitines and phospholipids, and impaired antioxidant capacity, collectively promoting oxidative damage and inflammatory signaling. The identification of these metabolome alterations might contribute to a deeper understanding of the toxicity and biological impact of environmental stressors on offspring health. These results may eventually lead to the identification of early biomarkers and to the development of therapeutic strategies aimed at reducing NCD risk.

## 1. Introduction

During pregnancy, the production of reactive oxygen species (ROS) and reactive nitrogen species (RNS) naturally increases as a result of enhanced metabolism, heightened placental mitochondrial activity, and elevated oxygen consumption. In order to ensure optimal fetal growth and development, the maternal body undergoes systemic adaptations that prioritize nutrient delivery to the fetus through regulation of amino acid, lipid, and energy metabolism, contributing to elevated ROS/RNS generation [1,2,3,4]. This physiological rise in ROS and RNS plays a crucial role in signaling pathways that are essential for proper embryogenesis, placental development, and vascular remodeling [5,6,7,8,9]. The maintenance of this sensitive dynamic redox balance is required to support healthy pregnancy. When the uncontrolled increase of ROS and RNS exceeds the capacity of antioxidant defense, oxidative damage of nucleic acids, lipids, and proteins can occur [10]. Such molecular damage contributes to dysfunction of placenta and related pregnancy complications such as fetal growth restriction, preterm birth, and small-for-gestational-age or large-for-gestational-age delivery [11,12,13,14,15,16]. Moreover, suboptimal fetal development may increase susceptibility to various diseases that may become evident later in life [17,18,19,20].

Exposure to environmental pollutants, including toxic metals, particulate matter, nitrogen oxides, and tobacco and nicotine-containing products, can further exacerbate oxidative stress and metabolic dysregulation in maternal–placental–fetal units, altering pathways such as lipid peroxidation, amino acid metabolism, and mitochondrial energy production, which are critical for nutrient transport and fetal growth [10,21,22]. These metabolic perturbations reflect alterations in cellular homeostasis that can manifest as measurable changes in specific maternal and fetal metabolites, termed metabolome [23,24,25]. As the metabolome reflects the end products of endogenous biological processes influenced by both genetic and environmental factors, it can serve as a valuable tool for investigating how various exposures contribute to diseases development. Metabolomics, the comprehensive analysis of these metabolites and their dynamic fluctuations within biological systems, enables detailed characterization of metabolic alterations at the time of sampling. This approach links environmental exposure to internal dose, physiological response, and the underlying mechanisms of disease development [26,27]. An overview of key metabolomics terminology is provided in the Glossary (Table A1) in Appendix A.

Recently, an increasing number of studies have investigated how environmental stressors modify alterations in the maternal–fetal metabolome during pregnancy. These studies often highlight changes in primary metabolic pathways, such as glycolysis, aerobic respiration, the citric acid cycle, fatty acid oxidation, and gluconeogenesis, which are essential for energy production and metabolic homeostasis. Disruption of these pathways may reflect dynamic interactions between cellular redox imbalance, mitochondrial dysfunction and external stressors, ultimately contributing to long-term disease risk [28]. Using different high-throughput analytical techniques such as NMR spectroscopy and mass spectrometry in a variety of biospecimens, metabolomics enables the identification and quantification of redox sensitive metabolites [29,30], providing a valuable insight into the biological impact of prenatal environmental exposures. This approach offers the potential to identify biomarkers indicative of oxidative damage and adaptive responses.

This narrative review aims to summarize recent epidemiological studies published since 2016 regarding the metabolomics response to prenatal exposure to metals, air pollution, and tobacco and nicotine-containing products in maternal–placental–fetal compartments. The primary focus is on exposure-related metabolome alterations related to oxidative stress pathways. A better understanding of these metabolomic responses may help clarify how environmental exposures to these pollutants disrupt metabolic homeostasis and contribute to oxidative stress-related disease mechanisms.

## 2. A Short Overview of Oxidative Stress in Normal (Healthy) Pregnancy

In a healthy pregnancy, oxidative stress results from increased metabolic demands and dynamic oxygen fluctuations that are essential for fetal development. During early gestation, the hypoxic environment of the placental bed supports embryogenesis and trophoblast invasion. At this stage, ROS generation is minimal to protect cells against teratogenic effects [15,31]. As pregnancy progresses, the development of uteroplacental circulation raises oxygen tension. This drives mitochondrial electron transport activity in placental cells and elevates ROS production [32]. The oxidative environment is counterbalanced by a coordinated activation of antioxidant defenses including glutathione (GSH), superoxide dismutase (SOD), glutathione peroxidase (GPx), and catalase (CAT). These antioxidants help neutralize excess ROS to prevent oxidative damage. At the same time, they allow ROS to continue their important roles in redox signaling processes essential for placental vascular remodeling, angiogenesis, and immune cells activation [31,32,33].

The interplay between ROS and antioxidants evolves across trimesters, reflecting the metabolic adaptations that occur through gestation. During the second trimester, rising maternal oxygen consumption and lipid catabolism increase hydrogen peroxide generation. Insulin resistance and fatty acid mobilization further amplify ROS production during the third trimester [31,32]. Placental mitochondria generate superoxide anions that participate in signaling pathways necessary for vascular remodeling and syncytiotrophoblast differentiation [15,34]. Clinical studies demonstrate that physiological oxidative stress peaks near term, when placental tissue and maternal serum exhibit the highest levels of antioxidant enzymes [32,35]. This delicate balance ensures that ROS contribute to vital processes such as cervical ripening and prostaglandin synthesis without causing pathological inflammation or DNA damage [36]. However, environmental and lifestyle stressors can disrupt this balance.

## 3. Metabolic Pathways in Redox Regulation During Pregnancy

Pregnancy involves substantial metabolic adaptations in protein, carbohydrate, and lipid metabolism to meet the continuous demand for nutrients and other substrates essential for placental development and fetal growth. These changes are necessary for creating an optimal intrauterine environment while simultaneously supporting the increased physiological demands of the mother [37]. Tightly regulated changes in these pathways help maintain energy balance and redox homeostasis through gestation, as each plays a key role in both the generation and regulation of ROS.

Carbohydrate metabolism adapts by increasing maternal glucose production and inducing insulin resistance, which helps to direct more glucose to the fetus [38,39]. Dysregulation of glycolysis or mitochondrial dysfunction in trophoblasts can reduce ATP production, impair nutrient transport, and increase ROS production, contributing to conditions like gestational diabetes mellitus [40], and preeclampsia [41]. Carbohydrate metabolism, including the pentose phosphate pathway, is also critical for generating NADPH that is essential for the regeneration of GSH and maintaining cellular redox balance [28].

Lipids and fatty acids are sources of energy, structural components, and signaling molecules [22,42]. The lipid profile varies considerably throughout pregnancy, from lipid synthesis and fat storage in early pregnancy to lipolysis and fatty acid oxidation later, generating ATP, but also ROS, particularly in the presence of polyunsaturated fatty acids (PUFAs)like docosahexaenoic acid (DHA) and arachidonic acid (AA) [7,42,43].

Amino acids are essential for protein synthesis and organogenesis, vascular development, and antioxidant defense with GSH, a tripeptide composed of cysteine, glycine, and glutamate, tryptophan, and arginine metabolites playing key roles in redox homeostasis [4,7,41,44].

Disruption of these pathways, whether by environmental pollutants or poor lifestyle habits, compromises antioxidant defenses and increases oxidative stress, contributing to placental dysfunction and pregnancy complications. The following sections explore how environmental stressors drive these metabolomic alterations, providing insights into potential biomarkers and intervention strategies to mitigate oxidative stress.

## 4. Environmental Pollutants and Their Effects on Metabolomic Profiles and Pathways During Pregnancy

There is a growing concern about the impact of environmental pollutants and lifestyle factors on pregnancy outcomes. Some of them can induce or mediate the formation of oxidative stress, thereby disrupting key metabolic pathways such as energy, carbohydrate, lipid, and amino acid metabolism in the maternal–placental–fetal unit [23,24,25,45], increasing susceptibility to risk of maternal and fetal complications [19,20]. Understanding the mechanisms by which environmental stressors lead to oxidative stress and affect metabolic pathways in mother and her offspring is crucial for elucidating how these stressors modulate maternal–child health.

### 4.1. Metal/Metalloid Exposure

Exposure to metals such as cadmium, lead, mercury, and metalloids such as arsenic remains a significant global health concern due to their persistent presence in the environment and their documented associations with numerous adverse health outcomes [46,47]. Pregnant women are primarily exposed through the consumption of contaminated food, inhalation of active and passive tobacco smoke, and, in the case of inorganic arsenic, the use of contaminated groundwater. These toxic elements tend to bioaccumulate in the maternal body over a lifespan, leading to systemic toxicity and inducing adverse health effects, even at low-level exposure within environmentally relevant concentrations. Lead, arsenic, and mercury readily cross the placenta, thereby increasing the fetal body burden, while cadmium tends to accumulate in the placenta with limited transfer to the fetus, impairing function and nutrient transport [48,49,50,51].

These prenatal exposures have been associated with redox imbalance [9,45,52], impaired steroidogenesis [53], epigenetic changes [54,55], and adverse outcomes for the mother and her child [56,57]. While the adverse effects of exposure to high levels of toxic metals are well recognized, the impact of low-level metal exposure, particularly during pregnancy, remains insufficiently understood. Recently, several studies investigated effects of low-level metal(loid) exposure during pregnancy on metabolome alterations in mothers and their offspring. Table S1 summarizes these studies.

#### 4.1.1. Cadmium

Environmental cadmium exposure in Chinese pregnant women has been linked to alterations in urinary metabolites, indicating effects on oxidative stress and nephrotoxicity. A comparison of metabolomic profiles between women in the lowest and highest tertiles of urinary cadmium during the first trimester of pregnancy in Wuhan revealed increased levels of L-cystine, reflecting an adaptive upregulation of antioxidant defenses required for glutathione synthesis and elevated levels of L-tyrosine and its oxidized derivative dityrosine, indicating protein oxidation [58]. Decreased levels of histamine and uric acid were suggested to possibly reflect early nephrotoxic effects of cadmium, indicating alterations in purine metabolism and impaired renal reabsorption.

In another study of 103 pregnant women from Taiyuan, the combined evaluation of cadmium and zinc exposure identified 51 significantly differently expressed metabolites implicating alterations across several biochemical pathways [59]. Activation of amino acids pathways, including arginine and proline metabolism, important in nitric oxide production and redox balance, may link metal exposure to greater metabolic risks, such as obesity, hyperlipidemia, and hypertriglyceridemia. It was suggested that higher levels of bovinic acid, a conjugated linoleic acid known for its anti-inflammatory and anticancer properties, may be indicative of possible protective effects of zinc against oxidative stress and inflammation. Authors also highlighted octadecylamine, a long-chain fatty amine, as a potential biomarker for cadmium exposure and noted that its levels may decrease with zinc supplementation, possibly reflecting an antagonistic interaction between these metals [59].

#### 4.1.2. Lead

Metabolomic alterations associated with lead exposure were investigated in the Programming Research in Obesity, Growth, Environment, and Social Stressors (PROGRESS) cohort in Mexico City, Mexico, during third trimester of pregnancy [60]. Untargeted metabolomic analysis identified 31 serum metabolites significantly associated with blood lead levels, demonstrating significant effects of short-term lead exposure on metabolites classified as fatty acids and conjugates, amino acids and peptides, and purines. Changes in 2-hydroxybutyrate and metabolites from the alpha-linoleic and linoleic acid pathways point to possible links between recent lead exposure, disrupted glucose metabolism, increased risk for gestational diabetes, and potential effects on the maternal gut microbiome. Elevated bone (patella and tibia) lead in this study [60] was associated with higher betaine levels, suggesting an influence on methylation and epigenetic regulation. These findings highlight multiple pathways through which both acute and chronic lead exposure may adversely affect maternal and fetal health.

#### 4.1.3. Arsenic

Studies in pregnant women from China revealed alterations in urinary metabolomic profiles associated with low-level arsenic exposure, reflecting oxidative stress and potential liver and kidney metabolic disturbances [61,62]. A comparison of metabolomic profiles between women in the lowest and highest tertiles of urinary arsenic identified significant changes [61]. Elevated levels of two metabolites indicated enhanced leukotriene E4 (LTE4) production via the arachidonic acid pathway, while increased thiocysteine suggested dysregulation of thiol redox metabolism, consistent with the known ability of arsenic to bind to sulfhydryl groups, impairing protein folding and amplifying oxidative stress [63]. On the other hand, several antioxidant metabolites such as glutathione, cystathionine ketimine and 1-(beta-d-ribofuranosyl)-1,4-dihydronicotinamide were significantly decreased, probably due to their consumption in response to elevated ROS. Additionally, decreased LysoPC (14:0) and increased p-cresol glucuronide and vanillactic acid may reflect arsenic-induced metabolic dysfunction in the liver and kidneys [61].

Metabolic alterations associated with urinary arsenic species and their potential link to gestational diabetes mellitus (GDM) were examined using the meet-in-metabolite analysis (MIMA) strategy [62]. Key joint metabolites mediating these associations were thiosulfate and phosphoroselenoic acid, which are both essential for cysteine/selenocysteine biosynthesis, and pyridoxamine 5′-phosphate, an active form of vitamin B6. These metabolites are involved in purine and amino acid metabolism, one-carbon metabolism (OCM), and glycometabolism, related to antioxidant defense and arsenic methylation, which transforms inorganic arsenic into less toxic species, facilitating excretion and reducing toxicity [64]. The authors reported that a positive association of As^5+^ with these potentially “preventive” (protective) metabolites suggests As^5+^ may help attenuate the risk of GDM, while As^3+^ appears to contribute to GDM risk by impairing glucose regulation and disrupting purine metabolism and OCM pathways [62].

The impact of prenatal exposure to inorganic arsenic (iAs) on neonatal metabolite alterations was investigated in the Biomarkers of Exposure to ARsenic (BEAR) pregnancy cohort in Gómez Palacio, Mexico, by analyzing the cord serum metabolome of 50 neonates [65]. Multivariate analyses identified 10 cord serum metabolites significantly associated with maternal urinary levels of iAs and/or its metabolites, and 17 cord serum metabolites significantly associated with iAs and/or its metabolites in cord serum. Eight metabolites were significantly associated with both maternal exposure and biotransformation indicators in urine and neonatal cord serum measures of iAs and its metabolites, including glutamate, glycine, isoleucine, mannose, methionine, O-acethylcholine, taurine, and tyrosine. These metabolites demonstrated that prenatal arsenic exposure perturbs metabolism of amino acids, vitamin B6, and the citric acid cycle. For example, elevated maternal urinary arsenic correlated with disrupted energy metabolism (e.g., increased mannose and succinate) and amino acid metabolism (e.g., decreased tyrosine and isoleucine). Similarly, higher total arsenic in cord serum was significantly correlated with increased mannose, and decreased glutamate, isoleucine, methionine, O-acetylcholine and tyrosine, further implicating adverse effects of prenatal arsenic exposure on neonatal energy and amino acid metabolism.

#### 4.1.4. Metallomics—Effects of Metal Mixtures on the Metabolome

Metallomics, which examines multiple metals and metalloids simultaneously, offers a more realistic assessment of maternal exposure because pregnant women are usually exposed to complex metal mixtures rather than single elements. Since metals can interact with each other in synergistic, additive, or antagonistic ways [66], analyzing mixtures allows researchers to detect both cumulative and specific effects that may be overlooked in single-metal studies [67]. Recent metabolomics studies support this approach, linking metal mixtures to alterations in metabolic networks relevant to fetal development and maternal health.

A metabolome-wide association study (MWAS) within the Boston Birth Cohort (MA, USA) evaluated the impact of in utero exposure to lead, mercury, cadmium, selenium, and manganese on fetal metabolic programming [68]. Data from 670 mother–child pairs identified 25 cord blood metabolites, including xenobiotics, lipids, amino acids, and nucleotides that were significantly associated with at least one of these metals measured in maternal red blood cells (RBCs). The strong positive association between cadmium and nicotine metabolites, cotinine, and hydroxycotinine confirmed existing evidence identifying tobacco smoke as a major source of cadmium exposure. An inverse association between lead and piperine, a major component of black pepper with demonstrated antioxidant properties, indicates reduced antioxidant capacity and enhanced oxidative damage, consistent with experimental findings that piperine reduces insulin resistance, inflammation, and oxidative stress [69]. Mercury exposure was significantly associated with lipid metabolites in both positive and inverse directions, consistent with mercury’s suspected cardiotoxic effects mediated through inflammation, oxidative stress, and endothelial dysfunction [70,71].

Another US-based study performed within a subset of the Puerto Rico Testsite for Exploring Contamination Threats (PROTECT) cohort investigated association between maternal blood concentrations of multiple metals/metalloids and alterations in plasma lipidomic profiles in 83 pregnant women, of whom 23 experienced preterm birth [72]. Higher maternal manganese and zinc showed an inverse association with plasmenyl-phosphatidylethanolamine (PLPE), particularly species containing polyunsaturated fatty acids (PUFA) chains, while arsenic and mercury showed positive associations with PLPE and plasmenyl-phosphatidylcholine (PLPC), respectively. These findings are somewhat unexpected, given that PLPE and PLCE are key components of cell membranes with proposed antioxidant and anti-inflammatory functions. The authors note that the mechanisms underlying these associations remain unclear. However, they may reflect compensatory metabolic responses to metal-induced oxidative stress rather than direct protective effects, particularly in the case of arsenic and mercury. It is also important to note that this was a relatively small study, and samples were collected during the late second trimester (at approximately 26th weeks of gestation), when decomposition of lipid storage may occur, which may affect the observed lipidomic patterns.

Further study performed in the Nanjing cohort (China) related maternal metal exposure during the second trimester to birth outcomes in newborns [73]. Arsenic exposure was associated with reduced birth weight and shorter gestational age, whereas mercury exposure correlated with increased birth weight and longer gestation. Metabolomic analyses identified 55 metabolites significantly associated with exposure to arsenic, mercury, cadmium, and cobalt, implicating disruption in lipid, carbohydrate, and amino acid metabolism. Key altered metabolites included decreased levels of 2-hydroxycaproic acid and 7b-hydroxycholesterol (lipid metabolism), lower rhamnose and succinic acid (carbohydrate metabolism), and changes in amino acid metabolites such as 3-methyladenine, which was found to mediate the negative effects of arsenic on birth weight.

In addition, prenatal exposure to metals has been linked to impaired neurodevelopment in children. In a large Wuhan Healthy Baby Cohort (N = 1088), Wuhan, China, maternal urinary levels of eleven metals including manganese, nickel, aluminum, lead, cadmium, and arsenic measured in early pregnancy were associated with cognitive scores [74]. Metabolomics analysis identified perturbations in amino acid metabolism (particularly beta-alanine and histidine pathways), riboflavin metabolism, purine and pyrimidine metabolism, and lipid metabolism including phospholipids and sphingolipids. These pathways mediated the link between prenatal metal exposure and reduced neurodevelopmental scores, implicating oxidative stress and disrupted energy metabolism as key mechanisms. Carnosine, a neuroprotective dipeptide with antioxidant properties, and glutamine, critical for neurotransmitter cycling and ammonia homeostasis, were among key metabolites mediating neurotoxic effects. Additionally, enhanced riboflavin metabolism may partially mitigate cognitive impairments, reflecting compensatory antioxidant responses.

Similarly, studies in the PHIME and HERACLES European cohorts [75] demonstrated that prenatal exposure to metals, including mercury, lead, arsenic, manganese, and cadmium, perturbed mitochondrial respiration-related pathways such as glycolysis, gluconeogenesis, and the urea cycle. Dysregulation of amino acid metabolism, including pathways involved in neurotransmitter and nitric oxide synthesis (arginine, proline, histidine), as well as lipid metabolism pathways related to membrane integrity and myelination, were also identified. Dietary antioxidants such as lycopene from tomatoes and omega-3 fatty acids from fish appeared to mitigate these impacts.

Trimester-specific changes in metal exposure and amino acid metabolism were investigated in a longitudinal cohort of 232 healthy pregnant women from Wuhan, China [76]. The results demonstrated significant associations between urinary levels of seven metals (cadmium, cobalt, copper, cesium, manganese, thallium, and vanadium) and intermediates of arginine and proline, tryptophan, tyrosine, and lysine metabolism. Cadmium showed significant positive correlation with 2-Oxoarginine, N2-Succinyl-L-glutamic acid 5-semialdehyde, and N-Succinyl-L,L-2,6-diaminopimelate, and negative association with creatinine, indole, and N-Methyltryptamine. These findings underline oxidative stress and mitochondrial dysfunction as central mechanisms by which prenatal metal exposures impair child neurodevelopment, mediated through alterations in amino acid, lipid, carbohydrate, and energy metabolism detectable via metabolomics in urine.

Finally, a two-year prospective study from Nanjing, China, investigated the impact of prenatal exposure to multiple metals on the metabolic profile of pregnant women and examined how these exposures relate to the timing of primary tooth eruption and the number of teeth erupted by age one in their infants [77]. Higher maternal urinary cobalt concentrations were significantly associated with later eruption of primary teeth and a reduced number of teeth at age one. Using MWAS approach, glycine was identified as the key metabolite mediating these effects. Glycine, a non-essential amino acid with known anti-inflammatory and antioxidant properties, is essential for the formation of dentin and enamel. Thus, authors suggested that decreased glycine associated with elevated cobalt exposure may impair teeth development. Metabolomic pathway analysis supported perturbations in amino acid metabolism (particularly glycine, serine, and threonine metabolism), energy metabolism via citric acid cycle, and other pathways related to oxidative stress defense. Although cobalt is essential for human health, these findings highlight the importance of controlling cobalt exposure during pregnancy, particularly in regions where industrial activities such as smelting operations and widespread use of cobalt-containing compounds contribute to increased maternal exposure.

### 4.2. Air Pollution

Air pollution is a complex mixture of solid particles, liquid droplets, and gases suspended in the atmosphere. Air pollutants such as particulate matter (PM), carbon monoxide (CO), nitrogen oxides (NOx), ozone (O_3_), and sulfur dioxide (SO2) have been most strongly linked to public health risks. Variability in particle size, surface area, chemical composition, and presence of redox-active compounds contributes to differences in oxidative potential of air pollutants and their ability to induce oxidative stress and inflammation [78,79,80]. A major source of urban air pollution is traffic related air pollution (TRAP), a complex mix of gaseous and particulate matter emissions from motor vehicles, and the wear of brakes and tires. The following paragraphs and Table S2 summarize published findings on air pollution-associated metabolomic alterations in lipid, carbohydrate, energy, and amino acid metabolism, with a particular focus on pathways linked to oxidative stress and its potential impacts during pregnancy.

Several studies suggest that PM_2.5_ exposure during pregnancy disrupts energy metabolism, contributing to adverse birth outcomes, particularly preterm birth. In 52 pregnant women from Fuzhou, Fujian Province, China [81], relatively low-level PM_2.5_ exposure during early pregnancy was associated with mitochondrial dysfunction via oxidative phosphorylation pathway, implicating impaired energy metabolism, increased inflammatory responses and preterm birth risk. Similar findings were reported among African American women [82], with increased maternal serum levels of carnitine and adenosine triphosphate (ATP), and decreased levels of adenosine, along with disruptions in aromatic amino acid metabolism, affecting neurotransmitter synthesis, redox homeostasis, and immune regulation [83]. A positive association with lysophosphatidylethanolamine (lysoPE) (20:3) further indicated adverse effects on cell membrane integrity, lipid signaling and immune response [82].

Consistently, the Shanghai Maternal-Child Pairs Cohort prospective study identified the second and third trimesters as critical windows for PM_2.5_-induced placental toxicity [84]. Elevated PM_2.5_ exposure in non-smoking pregnant women were linked to fatty acids, bile acids, carbohydrate, and amino acids, reflecting compromised energy supply, oxidative stress and inflammation that impair placental and fetal development. All these findings suggest that PM_2.5_-induced metabolic and inflammatory disruptions are critical contributors to risks of premature delivery.

Research conducted in regions with high air pollution exposure has further investigated its effects on maternal and fetal health. In 160 mothers from California’s Central Valley, USA [85], first trimester traffic-related exposure to CO, NOx, and PM_2.5_ was associated with widespread serum metabolome disruption in mid-pregnancy. Air pollution exposure correlated with decreased levels of fatty acids such as linoleic acid and heptadecanoic acid, and amino acids such as methionine, cysteine, L-histidine, and serine. Pathway enrichment analysis revealed substantial alterations in lipid, eicosanoid, and carbohydrate metabolism, including glycolysis, gluconeogenesis, and the citric acid cycle.

Another study conducted in the same region further explored whether the impact of air pollution exposures on the maternal serum metabolome differs in mothers whose children later develop autism spectrum disorders (ASD) [86]. Compared to controls, mothers of ASD children had higher hypotaurine and lower valine and isoleucine intermediates, lysine, phenylalanine, and asparagine, which dysregulation may reflect increased oxidative stress and inflammatory response [87,88]. Impairment of lipid metabolism included changes in fatty acid metabolism, carnitine shuttle and bile acid synthesis, pathways crucial for mitochondrial production of energy. In addition, changes in glycolysis, gluconeogenesis, fructose and mannose, galactose and pyruvate metabolism, and the pentose phosphate pathway, indicated impaired energy production and redox balance. Increased levels of uric acid in mothers with ASD children further suggested a pro-inflammatory and oxidative environment potentially contributing to ASD development.

In addition, a study performed in Mexican cohort exposed to high ambient PM_2.5_ during early to mid-pregnancy revealed increased bile acids and amino acids, decreased fatty acids (including linoleic acid), and dysregulated glycerophospholipids, indicating disrupted adipogenesis, peroxisome proliferator-activated receptor (PPAR)-α signaling, glucagon-like peptide-1 (GLP-1), and incretin hormone pathways [89]. These changes suggested impaired energy metabolism, fatty acid oxidation, and glycemic control, likely originating from pregnancy-related inflammatory and metabolic adaptations.

Further studies show that maternal exposure to PM_2.5_ during mid-pregnancy disrupts metabolic and lipid pathways, particularly in women with overweight or hyperlipidemia. The Maternal and Developmental Risks from Environmental and Social Stressors (MADRES) pregnancy cohort study linked PM_2.5_ exposure during the second trimester to disruption in vitamin A and D_3_, tyrosine, pyrimidine and ascorbate-aldarate pathways, bile acid and C-21 steroid hormone biosynthesis (e.g., progesterone depletion) [24], contributing to placental dysfunction and fetal growth restriction. Authors suggested that overweight or obese women may be at greater risk to metabolic disturbances due to pre-existing redox imbalances.

Similarly, a lipidomic study in mother–newborn pairs from Beijing cohort, China [90], found that maternal PM_2.5_ exposure during the second trimester increased cord serum levels of several lipid species and inflammatory adipokines (e.g., angiopoietin-like 4 (ANGPTL4), insulin-like growth factor binding-protein 2 (IGFBP), interleukin IL-12p40, and tumor necrosis factor receptor II (NRF-RII)) in hyperlipidemic mothers. Perturbations in phosphatidylcholines, triacylglycerides, and phosphatidylinositols created a pro-oxidant environment linked to increasing risk of adverse birth outcomes and cardiovascular disease development in later life.

A neonatal metabolomics study in California, USA, further demonstrated disruptions in lipid metabolism pathways associated with maternal PM_2.5_ exposure during the third trimester [91]. Using neonatal blood spots, the study identified consistently affected pathways including fatty acid activation, biosynthesis, and metabolism, the carnitine shuttle, and glycerophospholipid metabolism. These disruptions point to the impairment of cell membrane integrity and energy production [21,22]. Elevated pro-inflammatory arachidonic acid (precursor to prostaglandin and leukotriene), and decreased anti-inflammatory linolenic acid reflected disrupted inflammatory signaling and redox balance, contributing to abnormal placental and uterine blood flow [92]. Alterations in metabolism of sulfur-containing amino acids methionine and cysteine further suggested impaired methylation and antioxidant defense.

Recent studies by Holzhausen et al. [93,94] identified distinct metabolic signatures of prenatal and early-life exposure to ambient air pollutants, particularly traffic-related PM_10_, PM_2.5_, NOx, and O_3_ in minority populations. In the Southern California Mother’s Milk Study (Latino cohort) [93], prenatal exposure was significantly associated with changes in vitamin B6, pyridine, tyrosine, fatty acid, and nucleotide metabolism at one month of age, while postnatal exposures affected histidine, bile acid, and energy metabolism throughout infancy showing sex-specific differences. Pathway enrichment analyses consistently implicated disturbances in amino acid metabolism, including cysteine, methionine, histidine, and tryptophan pathways, as well as lipid metabolic pathways such as fatty acid activation, biosynthesis, and glycerophospholipid metabolism. A complementary multicohort analysis of the Atlanta African American mother–newborn pairs (N = 205) and the Latino mother–child pairs (N = 122) found that higher maternal NOx exposure during pregnancy led to significant metabolic alterations in amino acid metabolism (e.g., isoleucine, phenylalanine, histidine, asparagine, and glutamic acid), lipid metabolism (including stearidonic acid, and sphinganine), and carbohydrate metabolism in neonates and infants [94]. Pathway analyses across both cohorts indicated a central role for methionine, cysteine, and tryptophan metabolism that are involved in redox-sensitive processes, and linked these alterations to oxidative stress and inflammatory pathways.

Together, these studies provide evidence that prenatal and early-life exposure to air pollutants, particularly components of traffic-related air pollution, induces persistent disturbances in metabolic pathways essential for maintaining redox balance, energy homeostasis, and cellular defense mechanisms during early development.

### 4.3. Exposure to Tobacco Smoke and E-Cigarette Vapor

Tobacco smoke is a complex aerosol mixture containing over 9500 chemicals, including thousands of toxic compounds such as nicotine, polycyclic aromatic hydrocarbons, volatile organic compounds, metals like cadmium and lead, nitrosamines, numerous oxygen-containing compounds, and free radicals [95,96]. These constituents contribute to the generation of ROS, and exacerbate oxidative stress in both pregnant women and their offspring by overwhelming endogenous antioxidant defenses, including key enzymes like superoxide dismutase and glutathione peroxidase [45,53]. This imbalance disrupts redox homeostasis and damages lipids, proteins, and DNA. Maternal tobacco smoking during pregnancy significantly increases the exposure of both mother and fetus to these toxic substances, many of which readily cross the placental barrier, alter steroid hormone levels [53], and directly affect placental and fetal development. Epidemiological and experimental studies link in utero and early-life tobacco smoke exposure to fetal growth restriction, preterm birth, and increased risk of metabolic, respiratory, cardiovascular, and neurodevelopmental disorders later in life [97,98]. Furthermore, second-hand tobacco smoke exposure during pregnancy has also been associated with adverse maternal and fetal health outcomes, underscoring the importance of comprehensive exposure assessment. Moreover, the increasing use of e-cigarettes and other electronic nicotine delivery systems has raised concerns regarding their potential health risks, particularly to vulnerable populations [99,100]. Despite perceptions that e-cigarettes are a safer alternative to conventional cigarettes, their aerosols contain nicotine as well as numerous toxic substances that may have adverse effects on health [101,102]. Recent evidence suggests that even secondhand exposure to e-cigarette vapor presents serious developmental and metabolic risks during critical windows such as pregnancy. Several metabolomic studies have investigated the metabolic perturbations associated with prenatal tobacco smoke exposure, highlighting alterations in lipid, carbohydrate, energy, and amino acid metabolism linked to oxidative stress pathways. Table S3 summarizes these studies.

In the Generation R Study cohort in Rotterdam, the Netherlands, cord blood metabolite profiles from neonates exposed to maternal smoking showed decreased levels of mono-unsaturated acyl-alkyl-phosphatidylcholines and lysophosphatidylcholines, implicating altered lipid metabolism [103]. Metabolic adaptations differed according to the duration of tobacco exposure, with the first trimester smoking only affecting neonatal metabolites involved in the citric acid cycle and oxidative stress pathways, whereas continuous smoking throughout pregnancy was linked to increased inflammation responses, activation of transulfuration pathway, and insulin resistance. Sex-specific differences in neonatal metabolic response to tobacco exposure were also noted, particularly affecting lipid metabolites in girls.

Similarly, targeted analysis in two multiethnic maternal–child cohorts from Tennessee, USA, the Pregnancy Risk Assessment Monitoring System (PRAMS, N = 8600) and the Infant Susceptibility to Pulmonary Infections and Asthma following RSV Exposure (INSPIRE, N = 1918), showed that smoking during third trimester was associated with higher concentrations of free carnitine, glycine, and leucine in newborns of smoking mothers then in control [104]. Smoking cessation during pregnancy contributed to lower metabolite concentrations, comparable to those observed in newborns of non-smoking mothers, suggesting reversibility of metabolic perturbations.

Assessment of tobacco exposure via nicotine-related metabolite measurements directly in biological samples in diverse cohorts has consistently revealed disruptions in metabolic pathways essential for urea cycle and citric acid cycle linking lipid, carbohydrate, amino acid, and energy metabolism to the toxic effects of tobacco smoke [105,106,107,108]. The European LINA (Lifestyle and Environmental Factors and their Influence on Newborn Allergy risk) study in 35 mother–child pairs from Leipzig, Germany, showed that maternal smoking, confirmed through questionnaires and urinary biomarkers resulted with opposite effects on lipid and amino acid metabolism in mothers and newborns [105]. Metabolites involved in lipid metabolism such as diacyl- and acyl-ether phosphatidylcholines and sphingomyelins were downregulated in smoking mothers and upregulated in their newborns, except acylcarnitines, which were decreased in exposed newborns. Amino acid metabolism was increased in smoking mothers but decreased in exposed newborns, particularly for glutamine and glycine. Decreased lipid-soluble antioxidant capacity in the serum of smoking mothers, indicated increased oxidative damage related to tobacco smoke.

Similarly, a study from Greenwood, SC, USA, used second-trimester maternal serum cotinine as a measure of low-level nicotine exposure to assess metabolic profiles in 65 mother–child pairs from karyotypically normal pregnancies [106]. The results demonstrated significant metabolic perturbations in amniotic fluid with dysregulated pathways including aspartate and asparagine metabolism, pyrimidine metabolism, urea/amino group metabolism, and arginine and proline metabolism. Additionally, analysis of maternal serum identified leukotriene metabolism, linoleates, and eicosapentaenoic acid derived metabolites as the most affected pathways, reflecting smoking-related increased activation of inflammatory processes. The study found that even low-level recent nicotine exposure, indicated by maternal serum cotinine levels below 2 ng/mL, was associated with distinct perturbations in both maternal and fetal metabolomes supporting the role of smoking in promotion of oxidative stress and inflammation.

In the African American cohort [107], metabolomic analysis of maternal serum (N = 105) confirmed perturbations in 47 metabolites that closely relate maternal urinary cotinine levels with preterm birth, and shorter gestational age during early (8–14 weeks gestation) and late (24–30 weeks gestation) pregnancy. Lipid metabolism showed significant alterations in fatty acid and glycerophospholipid pathways. Cotinine was positively associated with proinflammatory lysophosphatidylcholines (LysoPCs), as well as with 17-hydroxyprogesterone and acylcarnitine, involved in fatty acid oxidation, which indicated disrupted energy production and lipid metabolism in smokers. A positive association was found with glutamate, serine, choline, and taurine, amino acids that play roles in regulating oxidative stress, inflammation, and vascular function [108,109,110]. Cotinine-related changes in carbohydrate metabolism, including glucose-6-phosphate, suggest that tobacco smoke may impair glucose metabolism and tolerance during pregnancy, contributing to adverse outcomes [111].

A large population-based untargeted MWAS of newborn dried blood spots (N = 899) in California, USA, also linked maternal perinatal smoking, defined by self-report, and cotinine/hydroxycotinine levels in newborn blood to widespread perturbations in metabolomic profiles [112]. Key affected metabolic pathways included vitamin A (retinol) metabolism, tryptophan and kynurenine metabolism, and arachidonic acid metabolism. Disruption of these pathways was associated with inflammatory responses, oxidative stress, and biological mechanisms implicated in chronic diseases of the lung and central nervous system [113,114].

Extending concerns about tobacco smoke, recent study has also investigated an impact of second-hand exposure to e-cigarettes vapors on maternal metabolome alterations in an ongoing prospective longitudinal cohort, the New York University Children’s Health and Environment Study (NYU CHES) [115]. This small sample-size preliminary study, with exposure assessed via self-report of “living with someone who smoked e-cigarettes during pregnancy” revealed significant differences in maternal urinary metabolic profiles between exposed and unexposed groups, including alterations in lipid, fatty acid, and amino acid metabolism. Several lipid-related metabolites, such as palmitamide, glycerol trihexanoate, 2,4-undecadiene-8,10-diynoic acid isobutylamide were enriched in the exposed group, whereas levels of 1,2,3-propanetriol triacetate was lower compared to control group. Altered levels of sedoheptulose-7-phosphate, a metabolite in the pentose phosphate pathway essential for NADPH generation and redox homeostasis, indicate disruption in energy production and potential oxidative stress. Moreover, amino acid metabolism was also perturbed, as indicated by altered levels of N-acetylputrescine, and tryptophan pathway metabolites 5-methoxytryptophan and 5-hydroxy-L-tryptophan. Despite its limitations, this study highlights the importance of protecting pregnant women and other vulnerable groups from exposure to tobacco products, even in indirect, second-hand contexts.

**Figure 1:**
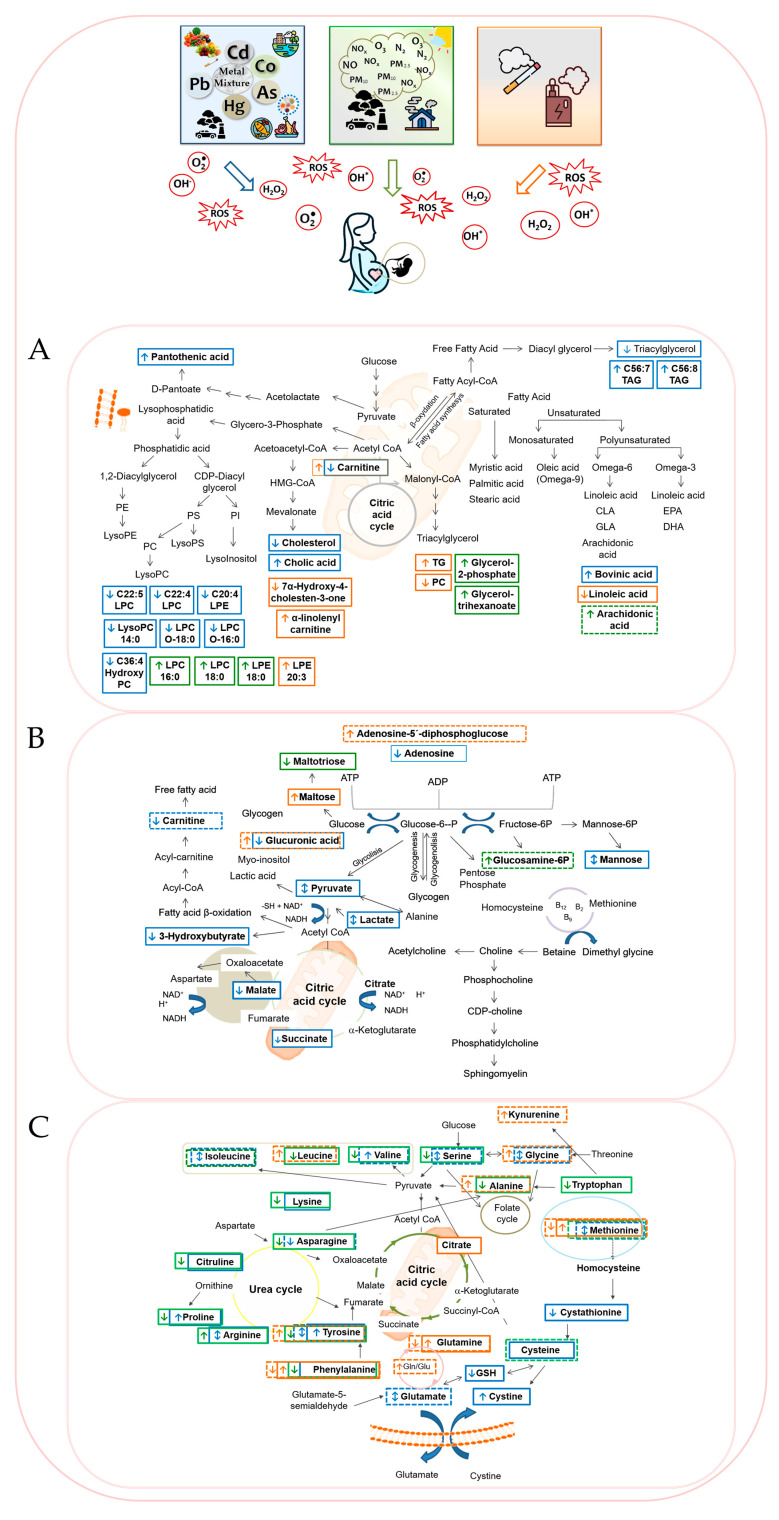
Integrated schematic overview of pollutant-related disruptions in (**A**). lipid, (**B**). carbohydrate, and (**C**). amino acid metabolism linked to oxidative stress. Exposure to metals/metalloid (blue), air pollution (green), and cigarette smoke/e-cigarette vapor (orange) are shown with their corresponding increases or decreases in specific metabolites. Maternal metabolome changes are marked with solid-line rectangles and offspring changes with dashed-line rectangles.

In summary, all these findings highlight that environmental pollutants, including metals, air pollution, and tobacco smoke and e-cigarette vapor, disrupt key metabolic pathways involving carbohydrates, lipids, and amino acids. These disruptions often lead to mitochondrial dysfunction and oxidative stress, which may mediate their adverse effects on maternal and fetal health. A schematic overview of key metabolic perturbations linked to pollutant exposure during pregnancy is illustrated in Figure 1.

## 5. Environmental Exposures and Metabolomic Disruptions in Maternal and Offspring Health

Maternal and fetal metabolism play a central role in maintaining health during pregnancy, operating as an integral network where redox balance, energy production, inflammation, hormonal signaling, and biosynthesis are intricately linked. Environmental pollutants have the potential to disrupt this delicate metabolic balance, as shown by metabolomic studies examining effects of toxic metals, air pollutants, and tobacco smoke and e-cigarette vapor. Maternal metabolic alterations, whether induced by metal mixtures, airborne pollutants, or nicotine, have direct consequences on fetal development. These changes alter nutrient transport, hormone levels (e.g., progesterone, bile acids, vitamins), and placental function, resulting in reduced fetal nutrient availability and compromised intrauterine growth [24,84]. It has been indicated that environmental exposure to toxic metals, air pollutants, and tobacco smoke commonly interferes with the body’s ability to control oxidative stress, regulate energy metabolism, and maintain optimal levels of amino acids, lipids, and carbohydrates [82,103,105].

Metals and metalloids like lead, cadmium, mercury, and arsenic have been shown in cohort and cross-sectional studies to disrupt key metabolic pathways related to amino acid metabolism (particularly arginine, valine, and methionine), lipid peroxidation, energy metabolism, and oxidative stress (Figure 1). These disruptions may be explained by the high affinity of these metals for sulfhydryl groups in amino acids, enzymes, and other biomolecules, which can impair protein function, enzyme activity, and antioxidant defenses [116,117]. Observed molecular perturbations have linked metal exposure in utero to impaired placental function, fetal growth restriction, and impaired neurodevelopment [58,60,62,74].

Similarly, air pollution exposure studies have documented metabolic alterations associated with PM_2.5_, NO_x_, and O_3_ with pathways involving oxidative phosphorylation, glutathione metabolism, and amino acid metabolism frequently affected [81,82] (Figure 1). Maternal serum and cord blood metabolomics reveal disruptions in lipid signaling molecules such as lysophosphatidylethanolamines and bile acids, implicating inflammation and oxidative stress in observed pregnancy complications such as low birth weight, small-for-gestational-age, and preterm birth [81,82,85,93]. These metabolic alterations likely reflect mitochondrial dysfunction and impaired antioxidant defenses [24].

In tobacco smoke exposure, metabolomic studies in maternal serum and neonatal blood spots show activation of detoxification pathways, increased oxidative stress markers, and alterations in carnitine and fatty acid metabolism [103,105,107] (Figure 1). Such perturbations correspond with risks for birth weight reductions and altered neonatal neurobehavioral outcomes [104]. In addition, nicotine exposure during gestation disrupts metabolic profiles in amniotic fluid associated with energy and amino acid pathways indicative of oxidative damage outcomes [106]. Emerging evidence also suggests that second-hand exposure to e-cigarette vapor may induce similar metabolic disruptions in homeostasis of redox-related metabolites in pregnant women, underscoring the need to further investigate the effects of presumed low-risk nicotine products [115].

Exposure-specific metabolite signatures, such as perturbed purine metabolism from metal exposure [60,62,74], disrupted bile acid and phospholipid profiles from PM_2.5_ [85,89], or altered tryptophan-kynurenine metabolism from tobacco smoke [106,112] underscore the mechanistic diversity of toxicant effects. These observed metabolomic alterations contribute to adverse pregnancy outcomes by inducing oxidative stress-mediated damage. Oxidative stress has been indicated as a key mechanism by which environmental pollutants disrupt placental nutrient and oxygen transfer, mitochondrial activity, and fetal metabolic programming. Disrupted amino acid metabolism and lipid peroxidation products reflect cellular injury and energy imbalance, potentially contributing to growth restrictions and neurodevelopmental impairments. Moreover, epigenetic modifications influenced by these metabolic changes may underlie the developmental origins of adult diseases. To illustrate these complex mechanisms, Table 1 summarizes the predominant metabolic pathways affected and associated health risks in mothers and offspring.

## 6. Strengths, Current Gaps and Challenges

Metabolomics enables comprehensive characterization of small-molecule metabolites that reflect physiological and pathological states in the body [118,119]. In toxicology, metabolomics facilitates the elucidation of mechanistic pathways through which pollutants induce biological effects, such as oxidative stress, inflammation, and metabolic dysregulation, particularly during sensitive windows like pregnancy [76,120].

Recent advances in high-resolution metabolomics have enabled the simultaneous measurement of thousands of metabolic features, providing a comprehensive view of the biochemical pathways affected by maternal environmental exposures. Both untargeted and targeted mass spectrometry-based approach have been widely applied to identify alterations in lipid, carbohydrate, energy, and amino acid metabolism, many of which are associated with oxidative stress processes. The integration of MWAS approach with pathway enrichment, bioinformatics tools, and existing biological knowledge has substantially advanced our understanding of exposure-related perturbations.

These achievements are particularly noteworthy given the inherent challenges of conducting research during pregnancy, including ethical considerations, limited access to suitable biospecimens, and the complexity of accurately assessing exposures that may occur before conception or during the earliest stages of gestation, when embryo is most vulnerable. Despite these barriers, researchers have made substantial progress in elucidating mechanisms underlying environmentally induced non-communicable diseases (NCDs), reflecting a strong commitment to advancing this complex area of study.

However, several important limitations remain [23,118,121]. Many studies use cross-sectional designs with relatively small sample sizes, sometimes fewer than 100 participants, and rely on single time-point exposure assessment. These design limitations make it difficult to determine cause-and-effect relationships and to capture dynamic changes in both exposure and metabolomic profiles over time. Accurate exposure assessment also remains a challenge. Metals are frequently measured directly in biospecimens such as blood, and urine, providing a direct measure of internal dose, integrating exposure from multiple sources and routes (ingestion, inhalation, dermal). These levels typically reflect only recent exposure, and may not capture long-term or cumulative exposure.

The placenta, although increasingly recognized as a valuable biomarker of in utero environmental exposure [122,123], remains underutilized in exposure assessment and is rarely incorporated in metabolomic studies. This limits the understanding of tissue-specific metabolic changes and the ability to directly link maternal exposures to fetal responses. Challenges related to placental sampling, biological variability, and analytical complexity likely contribute to its limited use in current metabolomic research.

Exposure to air pollution and tobacco smoke is typically estimated indirectly through external modeling or biomarkers with short half-lives, which increases the potential for exposure misclassification. Pollutant levels are often approximated using ambient air quality data modeled at residential locations or collected from fixed-site monitoring stations, failing to account for individual activity patterns and varying microenvironments, such as indoor versus outdoor settings or workplace exposures [124].

Most epidemiologic studies have focused on single pollutants, overlooking the reality of simultaneous exposure to multiple pollutants. However, an increasing number of metallomic studies have investigated the joint effects of multiple elements. This reflects a growing recognition of the need to capture the complexity of real-life scenario, in line with the exposome paradigm, which emphasizes the cumulative and interactive nature of environmental impacts on human health. Continued development of multi-pollutant approaches is essential for advancing comprehensive risk assessments and informing effective public health strategies.

Methodological heterogeneity across studies further complicates data interpretation and comparison. A persistent challenge is the limited availability of authentic chemical standards, which hampers confident metabolite identification and validation. Most cited studies used untargeted metabolomics that offers broad biochemical coverage, but provides only relative quantification, and is subject to variability in detection and limited reproducibility. Targeted metabolomics approaches allow for more precise quantification, typically supported by authentic standards, but are limited by preselection of metabolites. Liquid chromatography-mass spectrometry (LC-MS) remains the dominant platform due to its wide coverage of polar and semi-polar metabolites, while gas chromatography-mass spectrometry (GC-MS) retains indispensable utility for detecting volatile and thermally stable metabolites, providing complementary insights into metabolic alterations that LC-MS alone may overlook.

Overall, limited quantification accuracy and a lack of standardization across analytical platforms, methods and study designs make it difficult to replicate findings, identify reliable biomarkers or apply findings clinically. Ongoing efforts to overcome methodological barriers, along with continued technical advances and cross-disciplinary collaboration, are steadily refining our understanding and laying the foundation for future discoveries.

## 7. Opportunities and Future Directions

Recognizing these challenges, future studies should focus on improving exposure assessment methods and adopting longitudinal designs to capture critical windows throughout pregnancy [118,125]. Longitudinal sampling and data collection at multiple time points with repeated measures of exposure that begin before conception and follow participants throughout pregnancy into early childhood is essential to clarify the cause-and-effect relationships and their temporal dynamics. Expanding the use of biospecimens such as placenta, maternal and cord blood, will facilitate more precise exposure characterization, comprehensive biochemical profiling, and identification of tissue-specific mechanisms underlying adverse pregnancy outcomes. Investigation of sex-specific metabolic responses is also essential, as fetal sex may influence susceptibility to pollutant-induced metabolic disruption and shape both maternal and fetal adaptive responses. Paired sampling from maternal and fetal compartments can further enable direct comparisons and improve understanding of transplacental metabolic alterations.

Standardization and harmonization of metabolite identification and quantification protocols are essential to enhance reproducibility and comparability across studies [118,125]. Developing frameworks for absolute quantification and thorough method validation will foster biomarker utility in clinical and public health settings. Addressing real-world complex exposures requires multipollutant and exposome-wide approaches employing advanced statistical and computational tools to elucidate effects of chemical mixtures. Use of personal exposure monitoring technologies and refined exposure models that incorporate individual behaviors will reduce misclassification and better estimate internal dose. Integration of cutting-edge technologies such as artificial intelligence and systems biology will facilitate multidimensional data integration and novel hypothesis generation. Interdisciplinary collaboration across epidemiology, toxicology, biochemistry, and clinical sciences is crucial for accelerating progress and translating findings into preventive strategies. Although this review focuses on epidemiologic evidence of oxidative stress-related metabolomic alterations, these findings provide a foundation for future research exploring antioxidant-based prevention and therapeutic interventions to reduce exposure-related oxidative damage in vulnerable maternal–fetal populations. Leveraging these advances can deepen understanding of how prenatal exposures disrupt metabolic pathways through oxidative stress, ultimately improving maternal and fetal health outcomes. Ensuring adequate dietary intake, including diets rich in antioxidants and omega-3 fatty acids from diverse vegetables, fruits, fatty fish, and nuts, offers promising, practical opportunities to support maternal antioxidant defense and help mitigate oxidative stress, improve placental function, and decrease the risk of adverse pregnancy outcomes [4,126]. These dietary strategies could complement efforts to minimize environmental exposures. However, it is important to avoid excess nutritional interventions or supplementation that may carry risks of toxicity or contribute to oxidative stress [127,128].

## 8. Conclusions

Prenatal exposure to metals, air pollution, and tobacco smoke trigger a cascade of oxidative stress and metabolic dysfunction in both mother and fetus, contributing to adverse pregnancy outcomes and heightened early-life health risks. Metabolomic studies indicated that these exposures alter key biochemical pathways such as amino acid metabolism, lipid peroxidation, and mitochondrial energy production, compromising nutrient transport and redox balance. Specifically, toxic metals interfered with amino acid metabolism pathways involving glutamine, glycine, methionine, cysteine, and arginine, as well as impacting lipid and energy metabolism. Air pollution, especially PM_2.5_ and TRAP, was primarily linked to perturbations of lipid metabolism, including phospholipids and fatty acids, while tobacco smoke and e-cigarette vapor were associated with disturbances in mitochondrial dysfunction, lipid and bile acid signaling, and amino acid metabolism. These metabolic disturbances were linked to adverse pregnancy outcomes, including fetal growth restriction, preterm birth, and impaired neurodevelopment, and may influence long-term disease risk in offspring. Differences between mothers and offspring include somewhat opposing metabolomic responses, such as increased lipid metabolites in exposed newborns versus decreased in mothers, highlighting the value of paired biospecimen analyses.

Recognizing these findings, future studies should focus on improving exposure assessment, adopting longitudinal sampling to capture critical gestational windows, standardizing metabolomic approaches, and investigating sex-specific responses. The integration of multipollutant or exposome-wide modeling remains limited, but it is important for showing real-world exposure and clarifying interaction effects among chemicals. Mixed exposure scenario reflects real-life conditions but also presents analytical challenges that require sophisticated statistical and systems biology tools. Such advances will facilitate more accurate and reliable causal interpretation in environmental health outcomes and identify biomarkers predictive of fetal and child health risks. Given the substantial resources needed, it is essential that future funding mechanisms explicitly support and enable this type of comprehensive research.

## Figures and Tables

**Table 1:** Main metabolic pathways affected by metal, air pollution, tobacco smoke and e-cigarette vapor exposure during pregnancy.

Pollutant	Main Metabolic Pathways Affected	Health/Functional Implications	References
Metals (e.g., cadmium, lead, mercury, arsenic)	Disruption of amino acid metabolism (arginine, methionine, glycine, glutamine) Lipid peroxidation and altered membrane lipids (phospholipids, sphingolipids) Impaired energy metabolism (citric acid cycle, mitochondrial ATP production) Oxidative stress and mitochondrial dysfunction	Increased risk of neurodevelopmental impairment Fetal growth restriction Impaired placental function Long-term metabolic disease susceptibility	[58,59,60,61,62,65,68,72,73,74,75,76,77]
Air pollution (PM_2.5_, PM_10_, NO_x_, CO, O_3_)	Mitochondrial dysfunction, disruption of oxidative phosphorylation Lipid metabolism: altered fatty acids, phospholipids, bile acids Impaired amino acid metabolism (phenylalanine, tyrosine, tryptophan)	Increased preterm birth risk Low birth weight Placental insufficiency Neurodevelopmental impairment	[24,84,85,86,89,90,91,93,94]
Tobacco and e-cigarettes (maternal and secondhand)	Lipid signaling disruption: altered phosphatidylcholine, sphingomyelins (disrupted fatty acid oxidation) Disruption of amino acid metabolism (glycine, taurine, tryptophan) Reversibility observed in maternal metabolome upon smoking cessation	Oxidative stress-driven mitochondrial damage Disruption of cell membrane integrity Inflammation and vascular reactivity	[103,104,105,106,107,112,115]

## Data Availability

Not applicable.

## Supplementary Materials

The following supporting information can be downloaded at: https://www.mdpi.com/article/10.3390/antiox14121442/s1, Table S1: Overview of studies on metal/metalloid pollution-induced metabolomic changes in lipid, carbohydrate, and amino acid metabolism related to oxidative stress pathways; Table S2: Overview of studies on air pollution-induced metabolomic changes in lipid, carbohydrate, and amino acid metabolism related to oxidative stress pathways; Table S3: Overview of studies on tobacco smoke- and e-cigarette vapor-induced metabolomic changes in lipid, carbohydrate, and amino acid metabolism related to oxidative stress pathways.

## Abbreviations

The following abbreviations are used in this manuscript:

Asn	Asparagine
Asp	Aspartic acid
BEAR Cohort	Biomarkers of Exposure to Arsenic
CALINE4	The California Line Source Dispersion Model, version 4
EWAS	Environment, and Social stressors
FIA-MS/MS	Flow injection analysis coupled with tandem mass spectrometry
GDM	Gestational diabetes mellitus
Gln	Glutamine
Glu	Glutamic acid
HMDB	Human Metabolome Database
iAs	Inorganic arsenic
INSPIRE Cohort	Infant Susceptibility to Pulmonary Infections and Asthma following Respiratory Syncytial Virus (RSV) Exposure
KEGG	Kyoto Encyclopedia of Genes and Genomes
LC-HRMS	Liquid Chromatography—High Resolution Mass Spectrometry
LINA Study	Lifestyle and Environmental Factors and their Influence on Newborn Allergy Risk
LPA	Lysophosphatidic acid
LPC	Lysophosphatidylcholine
LPG	Lysophosphatidylglycerol
Lyso.PC.	Acyl-lysophosphatidylcholines
Lyso.PC.e	Alkyl-lysophosphatidylcholines
MADRES study	The Maternal and Developmental Risks from Environmental and Social Stressors
MIMA	Meet-in-metabolite-analysis
MITM	Meet-in-the-middle approach
MSE	Mass Spectrometry with Elevated Energy, untargeted fragmentation of all ions using alternating collision energies for wide metabolite coverage
MS/MS	Tandem mass spectrometry with targeted fragmentation of selected molecules to help identify compounds
MWAS	Metabolome-wide association approach
NIST	National Institute of Standards and Technology
PCaa	Diacyl-phosphatidylcholine
PCae	Acyl-alkyl-phosphatidylcholine
PHIME Cohort	Public Health Impact of long-term, low-level Mixed Element exposure in susceptible population strata
PRAMS	Tennessee Pregnancy Risk Assessment Monitoring System
PROGRESS Cohort	Programming Research in Obesity, Growth, Environment and Social Stressors
Pro	Proline
PROTECT Cohort	Puerto Rico Testsite for Exploring Contamination Threats
PTB	Pre-term birth
SM	Sphingomyelins
TAP data	Tracking Air Pollution in China (a combined geographic–statistical model utilizing satellite remote sensing data)
tAs	Total arsenic

## Appendix A

**Table A1:** Key Metabolomics Terminology Glossary.

**Metabolomic Features:**
Distinct metabolic signals or peaks detected by analytical platforms (e.g., mass spectrometry), representing putative metabolites that may be identified or unknown.
**Targeted Metabolomics:**
Quantitative analysis of a predefined set of known metabolites with high sensitivity, focused on specific biochemi-cal pathways or hypotheses.
**Untargeted Metabolomics:**
Global, unbiased profiling aiming to detect as many metabolites as possible, enabling discovery of novel metabolic changes and pathways.
**Pathway Enrichment Analysis:**
Statistical methods that identify biochemical pathways significantly impacted based on altered metabolite profiles, linking molecular changes to biological functions.
**Mummichog-based Metabolic Pathway Analysis:**
A computational strategy enabling pathway-level interpretation from untargeted metabolomic data without re-quiring full metabolite identification, facilitating rapid biological insights.
**Metabolome-wide association study (MWAS) approach:**
Systemic, high-throughput analysis that assesses associations between a broad spectrum of metabolite profiles and phenotypes, exposures, or disease outcomes, enabling unbiased identification of metabolic biomarkers or pathways linked to health and disease risk.
**Meet-in-metabolite-analysis (MIMA):**
A strategy used to reconstitute the adverse outcome pathways (AOPs) by identifying metabolomic linkages be-tween environmental exposures and associated health outcomes or diseases.
**Meet-in-the-middle approach (MITM):**
A research method that identifies biological markers linking environmental exposure to disease by finding meta-bolic changes associated with both the exposure and the health outcome.
**Exposome-wide association study (EWAS):**
A research approach that systematically screens a large number of environmental exposures to identify which are statistically linked to specific health outcome, similar to how genome-wide association studies identify genetic as-sociations.

Note: Definitions in this glossary were adapted from [129,130,131].
