# Supplementary material for: Oxidative Stress-Related Metabolomic Alterations in Pregnancy: Evidence from Exposure to Air Pollution, Metals/Metalloid, and Tobacco Smoke
Source: Antioxidants (Basel). 2025 Nov 30;14(12):1442. doi: 10.3390/antiox14121442 (PMC12730112; doi:10.3390/antiox14121442)
Supplement: Supplementary file 1 [file antioxidants-14-01442-s001.zip › antioxidants-3959193-supplementary.pdf]

Oxidative Stress-Related Metabolomic Alterations in Pregnancy: Evidence from Exposure to Air Pollution, Metals/Metalloid, and Tobacco Smoke

Alica Pizent

Supplementary Materials

Table S1. Overview of studies on metal/metalloid pollution-associated metabolomic changes in lipid, carbohydrate, and amino acid metabolism related to oxidative stress pathways.

Exposure	Population	Metabolomic Method/Metabolite Identification	Lipid Metabolism	Carbohydrate Metabolism	Amino Acid Metabolism	Reference
Cadmium (Cd)						
Low-level environmental exposure, assessed via urinary Cd (mean range: 0.3-1.1 µg/g creatinine) at the end of the first trimester (11th to 13th week of pregnancy)	Pregnant women (N=246) from Wuhan, China, in first trimester (2013 to 2014). Cross-sectional study	Untargeted analysis via UPLC/Q-TOF MS. Accurate *m/z* and fragment ions via MS^E. HMBD and METLIN database. Mass error tolerance: 10 ppm. Confirmation by comparison with commercial standards.	Maternal urinary metabolome			[58]
			N/A	N/A	↑L-cystine (precursor for glutathione synthesis), ↑L-tyrosine (oxidative modification), ↑Dityrosine (marker of protein oxidation) ↓Histamine	
Cd and zinc (Zn) in maternal urine in first trimester (≤ 14 weeks). Groups defined by median splits: LZnLCd, HZnLCd, LZnHCd, HZnHCd. Median (IQR)	Pregnant women (N=185) from Taiyuan, China, in first trimester (2018 to 2020). Cross-sectional study.	Untargeted UPLC-MS analysis. Internal MS2 database (BiotreeDB), HDBM	Maternal urinary metabolome (N=103)			[59]
			Group with higher Cd: ↑Pantothenic acid ↑Octadecylamine ↑Bovinic acid	N/A	↑Arginine and proline metabolism, ↑Arginine and histidine metabolism	

Cd 0.675 (0.457, 0.998)<br>Zn 2714 (2062, 3410)						
Lead (Pb)						
			Maternal serum			[60]
Environmental exposure in third trimester assessed via blood Pb: median (range) 29 (3-145) µg/L	Pregnant women (N=99) from Mexico City, Mexico, in third trimester (July 2007 to February 2011), the PROGRESS cohort.	Untargeted analysis via UHPLC-Q-TOF MS. MWAS approach, in-house database matching considering retention time, accurate mass, and MS/MS matching with pure standards (when available) (based on Metabolomic Standard Initiative criteria)	↑1-arachidonoylglycerol<br>↑glycochenodeoxy-cholic acid<br>↑Glycocholic acid<br>(fatty acids, bile acids)	↓2-hydroxybutyrate<br>↓metabolites in α-linolenic and linolenic acid pathway	↓5-aminopentanoic acid<br>↓L-arginine<br>(alterations in amino acids and peptides)	
.... via bone Pb median (IQR)<br>Tibia Pb=2.5 (7.3) µg/g,<br>Patella Pb=3.6 (9.5) µg/g.	Pregnant women (N=89) from Mexico City, Mexico, in third trimester (July 2007 to February 2011), the PROGRESS cohort.		↑1-arachidonoylglycerol<br>↑Glycochenodeoxy-cholic acid<br>↑Glycocholic acid<br>(fatty acids, bile acids)	N/A	↑Betaine<br>(methylation, possible gut microbiome involvement)<br>↓5-aminopentanoic acid<br>↓L-arginine<br>(alterations in amino acids and peptides)	
Arsenic (As)						
			Maternal urinary metabolome			[61]
Low-level environmental exposure, assessed via urinary As in the first trimester (11th to 13th week of pregnancy).<br>Low As group: 15.6±2.9 µg/g creatinine,<br>Middle As group: 23.6 ±2.4 µg/g creatinine,<br>High As group: 49.9±32.7 µg/g	Pregnant women (N=246) from Wuhan, China, in 11th to 13th week of pregnancy (November 2013 to May 2014). Cross-sectional study	Untargeted analysis via UPLC/Q-TOF MS. m/z matched with the in-house localized HMBD-derived database. Comparison of retention time with standards (GSH, p-cresol glucuronide, and vanillactic acid), others by MS/MS fragmentation. Mass error tolerance: 10 ppm	↑18-carboxy-dinor-LTE4,<br>↑20-COOH-LTE4<br>(increased lipid peroxidation, membrane phospholipid cleavage)<br>↓LysoPC (14:0) (liver and kidneys metabolic disorders)	↓1-(beta-d-ribofuranosyl)-1,4-dihydronicotinamide (NAD(P)H precursor)	↓ Glutathione;<br>↓Cystathionine<br>↓Ketimine<br>(intermediates in the pathway methionine to cysteine);<br>↑Thiocysteine<br>(desulfuration of cysteine)	

creatinine.						
Environmental exposure during 24th to 28th gestational week.<br>As-species (As^{3+}, As^{5+}, MMA, DMA, AsB) in maternal urine. Total urine As classified into quintiles. Median specific gravity-adjusted concentration of iAs=2.76 µg/L, tAs=21.64 µg/L.	Pregnant women (N=399, of whom 91 with GDM) from Tianjin, China, at 24th to 28th gestational week (October 2017 to January 2018).<br>Cross-sectional study	Untargeted analysis via UPLC/MS/MS.<br>MIMA approach.<br>HMBD search based on accurate mass.<br>Mass error tolerance: 10 ppm	Maternal urinary metabolome			[62]
			Related to As^{3+}:<br>↑Cholic acid (primary bile acid produced in the liver)	Associated with DMA and tAs:<br>↑D-glucose	Related to As^{3+}:<br>↑Thiosulfate,<br>↓Dihydrobiopterin,<br>↓Phosphoroselenoic acid<br>↑Acetylcysteine (cysteine/selenocysteine biosynthesis, related to One-Carbon-Metabolism)<br>↓ Pyridoxamine 5′-phosphate (vitamin B6 active form);<br>↑Histidylhistidine	
Environmental exposure based on both environmental samples (drinking water) and biological samples (maternal spot urine and cord serum), with quantification of iAs and its metabolites (at the time of delivery). Maternal mean urinary tAs=61.4 µg/L iAs=3.4 µg/L and neonatal cord serum tAs=610 pg/L, iAs=55 pg/L.<br>iAs in the water from this area ranges up to 236 ppb (literature data)	Neonates (N=50) from the BEAR Cohort from Gómez Palacio, Mexico, with mothers recruited before delivery (August 2011 to March 2012).	Targeted analysis via ^{1}H NMR Spectroscopy.<br>Relative quantification.<br>Metabolites identified and quantified using Chenomx NMR Suite software with library (36 metabolites matched)	Neonatal cord serum			[65]
			Associated with As-species (direction varies):<br>Acetoacetate<br>Glycerol<br>Betaine<br>3-hydroxybutirate	Associated with As-species (direction varies):<br>Mannose<br>Pyruvate<br>Lactate;<br>Succinate (energy metabolism)	Associated with As-species (direction varies):<br>Glutamate<br>Methionine<br>Isoleucine<br>Valine<br>Tyrosine<br>Serine<br>Glycine	

Metallomics						
Maternal exposure to Pb, Hg, Cd, Se, and Mn measured in maternal red blood cells (RBCs) collected 24-72 h after delivery	Mother–child pairs (N=670) from the Boston Birth Cohort, USA, a racially and ethnically diverse urban cohort, recruited after delivery (between December 2002 and October 2013).	Targeted analysis via LC-MS. MWAS approach was used. 378 known metabolites were identified.	Cord blood			
			Hg:: ↓C22:5, ↓CE, ↓C22:4 LPC, ↓C22:5 LPC, ↓C20:4 LPE, ↑C56:7 TAG, ↑C56:8 TAG, ↑C58:11 TAG, and ↑C60:12 TAG. Mn: ↑C36:4 hydroxy-PC Se: ↓C19:0 LPE B	Se: ↓C6 carnitine ↓C7 carnitine (both involved in energy metabolism)	Se and Mn: ↓Guanidinoacetic acid (GAA) ↓Imidazole propionate (ImP) Se: ↓Asparagine ↓Hydroxyproline	[68]
Maternal levels of 16 metals and metalloids in blood collected at 26 weeks of gestation, including As, Cd, Cr, Cu, Co, Fe, Hg, Mn, Zn.	Pregnant women (N=83) from the Puerto Rico PROTECT cohort, (USA) at 26 weeks of gestation (2010-2016), including 23 with preterm birth and 60 term birth controls.	Targeted lipidomics via HPLC-MS. In total, 587 lipid species across 19 classes were identified with LipidBlast library and quantified using Multiquant and normalized to internal standards.	Maternal plasma			
			Ni: ↑LysoPC Mn and Se: ↓polyunsaturated plasmenyl-phosphatidylethano-lamine (PLPE) As: ↑PLPE Hg: ↑ plasmenyl-phosphatidylcholine (PLPC)	N/A	N/A	[72]
Maternal levels of 15 metals and metalloids including As, Cd, Co, Hg, Pb, Zn in serum in the second trimester.	Mother–child pairs (N=292) from Nanjing, China, at second trimester (2006-2011)	Untargeted analysis via UPLC HRMS. Exposome-wide association study (ExWAS) approach. Metabolite identified by comparison to authentic metabolite standards in a custom constructed library TraceFinder (the	Maternal serum			
			Hg: ↓Cholesterol ↓2-hydroxycaproic acid ↑erucic acid Co: ↓L-carnitine	Hg: ↓D-glucuronic acid Cd: ↓Succinic acid Co: ↓L-malic acid ↓Rhamnose	Cd: ↓5-Hydroxylysine Co: ↓3-indolepropionic acid ↓Fumaric acid ↓Hippuric acid	[73]

		retention time, accurate mass, MS spectra) and relatively quantified with internal stable isotope-labeling internal standards.				
Maternal exposure to Al, As, Cd, Cr, Ga, Mn, Ni, Pb, Rb, Tl, V) during early pregnancy (<16 weeks) based on spot urinary levels at 13.1±1.1 weeks of gestation	Mother–child pairs (N=1088) from Wuhan Health Baby Cohort (WHBC), Wuhan, China, recruited at early pregnancy (13.1±1.1 weeks of gestation, from March 2014 to March 2016). Cord blood samples were collected at birth.	Targeted analysis (45 amino acids and its metabolites, 11 hormones, 9 carnitines, 7 bile acids, 7 vitamins) UPLC-QTRAP-MS. Untargeted lipidomics via UPLC-QTOF-MS/MS. MWAS and MITM approach. Polar lipids identification by using MS-DIAL, with mass tolerances/accuracy of 0.01 Da for MS and 0.05 Da for MS/MS.	Cord plasma			[74]
			Cr, Mn, Pb, Ga: ↓multiple Lysophospholipids (e.g., LPA 20:4, LPC O-16:0, LPC O-18:0, LPC O-18:1, LPC O-24:2, LPE 22:4, LPG 20:4)	N/A	Mn, Pb, Ni, V, Al, Ga: ↑Carnosine, ↑Valine, ↑Hydroxylysine, ↑Asymmetric dimethylarginine, ↑Targinine, ↓Glutamine<br>Cr, Mn, Ni, Tl, Al, Rb: ↑Riboflavin	
Environmental exposure to As, Cd,	Mother–child pairs to evaluate in utero and	Untargeted analyses with combined	Maternal urine and plasma			[75]

Hg, Pb, Mn, Cu, Zn, Se, Fe, Mg, and Ca based on its levels in various matrices collected at different time points: maternal blood, hair, urine and cord blood (collected around 34 weeks of gestation, at or immediately after birth), breast milk (one month postpartum).	earl-life exposure effects.<br>PHIME Cohort (N=133 pairs from Greece, Slovenia, Croatia, and Italy, enrolled 2006 to 2011).<br>HERACLES Cohort (N=300 children aged 3 to 8 years from Greece, near a waste disposal site). Child neurodevelopment assessed at 18 months (PHIME) and 3-8 years (HERACLES).	UPLC-HRMS and NMR platforms EWAS approach. Metabolite identification by using the ChenoMx NMR Suite, LipidMaps, and by comparing retention times and fragmentation patterns with those of authentic standards from an in-house library or MS/MS spectra from HMDB and Metlin databases.	↓metabolism of fatty acids<br>Carnitine<br>Acylcarnitines	Impaired energy metabolism. Impaired glycolysis/gluconeo-genesis.<br>Lactate, Pyruvate	Disrupted amino acid metabolism involved in neurotransmitter and nitric oxide synthesis (e.g., arginine, proline, and histidine pathway).<br>Disrupted urea cycle (e.g., L-arginine and L-citruline).<br>Alanine<br>Lysine	
Levels of 16 metals including Cd, Co, Cu, Cs, Mn, Tl, and V in urine of pregnant women in the first, second, and third trimester.	Healthy pregnant women (N=232) from Wuhan, China, recruited from November 2013 to July 2014 and followed throughout all trimesters. Urine samples were collected during the first, second, and third trimesters.	Targeted analysis via UPLC-Q-TOF-MS. Comparison with structural information in the HMDB, METLIN or MassBank databases.		Maternal urine		[76]
			N/A	N/A	Cd, Co, Cu, Cs, Mn, Tl, and V significantly correlated with: 2-oxoarginine, 3-indoleacetonitrile, indole, indole-5,6-quinone, N2-succinyl-L-glutamic acid 5-semialdehyde, N-methyltryptamine and N-succinyl-L,L-2,6-diaminopimelate	
Levels of 12 metals including As, Cd, Co, Cu, Fe, Se, Hg, Tl) measured in maternal urine collected during first	Mother–child pairs (N=183) from Nanjing, China, in their first trimester September 2014 to August 2015).	Targeted UPLC-HRMS analysis. Metabolite identified by commercial standards.		Maternal urine		[77]
			N/A	N/A	Glyoxylate and dicarboxylate metabolism, cyanoamino acid metabolism, citrate	

trimester.					cycle (TCA cycle), sphingolipid metabolism, aminoacyl-tRNA biosynthesis, methane metabolism, lysine degradation, and glycine, serine and threonine metabolism.	

Table S2. Overview of studies on air pollution-associated metabolomic changes in lipid, carbohydrate, and amino acid metabolism related to oxidative stress pathways.

Exposure	Population	Metabolomic Method/Metabolite Identification	Lipid Metabolism	Carbohydrate Metabolism	Amino Acid Metabolism	Reference
Ambient $PM_{2.5}$ components (NH_4^+, SO_4^{2-}, NO_3^-, organic matter, black carbon), TAP data. Relatively low-level exposure. During pregnancy.	Pregnant women (N=26 women with PTB + 26 healthy controls) from Fuzhou, Fujian Province, China. Early pregnancy.	Untargeted analysis via UPLC-MS/MS. In-house MS/MS database for annotation.		Maternal serum metabolome		[81]
				Oxidative phosphorylation		
Ambient $PM_{2.5}$ (one	Pregnant women	Untargeted analysis		Maternal serum metabolome		[82]

year prior to conception, the first trimester, and the one-month and one-week prior to blood sampling) via spatiotemporal modeling.	(N=330) from Atlanta, USA, in early pregnancy (6 to 17 week of gestation, March 2014 to May 2018).	via HR-LC-MS. 12 QC, NIST 1950. m/z, retention time, ion dissociation patterns to authentic chemical reference standards. Meet-in-the-Middle and High-dimensional mediation analyses.	↑Carnitine, ↑LysoPE(20:3) (Fatty acid metabolism)	↓Adenosine ↑ATP (Oxidative phosphorylation, energy metabolism)	Phenylalanine, ↓Citruline ↓Leucine ↓Proline ↓Tyrosine, ↓Tryptophan (protein digestion and absorption)	
Ambient exposure to $PM_{2.5}$ (assessed by both 72h individual air samplers and satellite-based models). During pregnancy (three trimesters, with focus on 2nd and 3rd trimester)	Pregnant women (N=329) from Shanghai, China, in third trimester (February 2017 to October 2018). No active smoking and drinking history.	Untargeted analysis via UHPLC-MS/MS. In-house and literature sources, quality control, Meet-in-the-Middle	Maternal serum metabolome			[84]
			↑Caprylic acid (CA, a medium-chain fatty acid) and ↑Tauroursodeoxy-cholic acid (TUDCA, a bile acid). CA and TUDCA positively correlated with $PM_{2.5}$ but inversely with chorionic placental disk area	↓Maltotriose (MT, a carbohydrate metabolite) associated with both $PM_{2.5}$ and placental changes	↑L-arginine, ↓Glycylproline and ↓N-acetylneuraminic acid (NANA, a sialic acid derivative) with $PM_{2.5}$ but in opposite direction with chorionic placental disk area	
Traffic related air pollution ($PM_{2.5}$, NOx, CO) via CALINE4 based on residential addresses, assessed in first trimester. High to low exposure levels.	Pregnant women (N=160, of whom 98 "highly exposed", and 62 "low exposed") from California's Central Valley, USA, during mid-pregnancy (around 16th weeks gestation) (California birth records, children born between 2005 and	Untargeted analysis via LC-HR-MS. Matching peaks by accurate mass and retention time to authentic reference standards in an in-house library.	Maternal serum metabolome			
			Disrupted fatty acid metabolism: ↓linoleic acid, ↓heptadecanoic acid, altered phospholipid, linoleate, leukotriene, and prostaglandin pathways.	Altered glycolysis, gluconeogenesis, and TCA cycle (energy metabolism)	↓Serine, ↓L-histidine, ↓(methionine, cysteine, and histidine pathway)	[85]

	2010).					
Traffic related air pollution ($PM_{2.5}$, NOx, CO), high exposure levels CALINE4 based on residential addresses. First trimester.	Pregnant women (N=214: 116 had offspring later diagnosed with autism spectrum disorder, 98 had neurotypical children) from California's Central Valley, USA, PROTECT Cohort. Mid-pregnancy.	Untargeted analysis via LC-HR-MS. Identification by using xMSannotator, NIST 1950, and HMDB, by matching the accurate m/z and retention time to authentic chemical standards, KEGG, LipidMaps, mass error threshold 10 ppm	Maternal serum metabolome			[86]
			↓L-carnitine ↑7α-Hydroxy-4-cholesten-3-one (7α-OH-4-CA) ↑alpha-linolenyl carnitine, ↑tetracosapentaenoyl carnitine.	↓3-Hydroxybutanic acid ↑Glycolate	↑Hypotaurine ↓Phenylalanine ↓Valine, ↓Lysine, ↓Taurine, ↓Asparagine, and ↓Alanine	
Ambient exposure to $PM_{2.5}$ assessed using high-resolution satellite-based machine learning models. High-exposure levels exposure during early- to mid-pregnancy.	Pregnant women (N=544) from Mexico City, Mexico (PROGRESS Cohort), recruited during second trimester (2007-2011). Maternal serum was collected at 1 month postpartum.	Untargeted analysis via LC-HRMS-QTOF-MS Identification by authentic reference library of standards MWAS approach	Maternal serum			[89]
			↑Bile acids, disrupted fatty acid metabolism (↓Linoleic acid metabolites and derivatives), dysregulated glycerophospholi-pids (most up)	Impaired energy metabolism (malate-aspartate shuttle) and glycemic control	↑N-formyl-L-methionine ↑proline ↑alanine ↑pantothenic acid Altered taurine metabolism.	
Ambient $PM_{2.5}$, PM_{10}, NO_2, and O_3 (US EPA Air Quality System), residential 24h averaged daily levels: trimester-specific pollutant concentrations for the preconception period, and the first and second	Pregnant women (N=382) from Los Angeles, CA, USA (MADRES study), predominantly lower-income Hispanic women (enrolled between 2015 and 2020). Fasting serum metabolome at the third trimester.	Untargeted analysis via LC-HRMS. MWAS analysis. Identification based on MS/MS fragmentation and retention time of authentic standard.	Maternal serum			[24]
			Disrupted C21-steroid hormone biosynthesis and metabolism, bile acid biosynthesis, fatty acid activation, glycosphingolipid biosynthesis, vitamin D3 and A	Altered glycolysis, gluconeogenesis, ascorbate (vitamin C) and aldarate metabolism	Altered tyrosine metabolism and other amino acid pathways	

trimester.	Subgroups formed by pre-pregnancy BMI, “Underweight or normal” vs. “Overweight or obese”.		metabolism, altered eicosapentaenoic acid (FA 20:5)			
Ambient exposure to $PM_{2.5}$ at ground level (the China National Environmental Monitoring Center) during the second trimester. A random forest machine learning model was applied per mother for each pregnancy period.	Mother–newborn pairs (N=203: 90 with hyperlipidemia in early pregnancy, 113 controls) from Beijing, China, enrolled between February 2017 and October 2018. Cord blood lipidome and adipokines.	Pseudotargeted lipidomics via LC-MS/MS. Authentic standards for identification, internal standards for quantification.	Cord blood			[90]
			Altered lipid species (10 different lipid classes) and adipokines in cord blood: PC (mostly ↓) TG (mostly ↑).	N/A	N/A	
Ambient exposure to traffic related air pollution ($PM_{2.5}$) during pregnancy (3rd trimester, modeled via CALINE4), local emissions.	Newborns (dried blood spots, N=241) from healthy infants, California, USA, born between 1998 and 2007. California Biobank	Untargeted analysis via LC-HRMS. Identification based on: matching to an authentic chemical standard, with a mass error threshold: ± 5 ppm, and retention time error: ± 15 s; matching to HMDB database, with a mass error threshold: ± 10 ppm.	Neonatal dried blood spots			[91]
			Altered lipid metabolism, including changes in fatty acids (activation, de novo biosynthesis, and metabolism), carnitine shuttle and glycerophospholipid metabolism; pathways linked to inflammation	Disrupted glycolysis and gluconeogenesis; changes in energy metabolism pathways	Altered amino acid metabolism including branched-chain and sulfur amino acids (methionine and cysteine metabolism)	
Ambient exposure to PM_{10}, $PM_{2.5}$, NO_2, and O_3 was modeled based on residential addresses to assess prenatal and	Mother-infant pairs (N=127), Latino birth cohort from the Southern California Mother’s Milk study, enrolled between 2016	Untargeted via LC-HRMS. Identification confirmed by matching authentic standards based on	Infant stool			[93]
			Prenatal exposure to PM_{10}, $PM_{2.5}$, and NO_2 was associated with fatty acid activation, fatty acid	N/A	Prenatal exposure to PM_{10}, $PM_{2.5}$, and NO_2 was associated with cysteine and methionine	

postnatal exposure.	and 2019, Los Angeles, CA, USA, followed at 1-, 6-, 12-, 18-, and 24-months postpartum.	m/z and retention time with a mass error threshold: ± 10 ppm and ± 50 s.	biosynthesis, and glycerophospholipid metabolism. Postnatal exposure to NO_2 was associated with arachidonic acid metabolism		metabolism, histidine metabolism, and tryptophan metabolism, ↓levels of metabolites involved in tyrosine metabolism.	
Prenatal exposure to ambient NOx (a major component of traffic-related air pollution), based on residential exposure using R-LINE and CMAQ models, during the first, second, and third trimester.	Mother-infant pairs from two independent cohorts located in geographically distinct regions: the prospective Atlanta African American Cohort (AAA, N=205) and the Southern California Mother's Milk Study (MMS, N=122). Dried blood spot samples collected within 24 and 48 h after birth and infant stool at 1 month of age.	Untargeted analysis via UHPLC-HRMS. Peak identification and alignment by Progenesis QI. Identification based on matching to an authentic chemical standard (m/z and retention time within 10 ppm and 50 s).	Neonatal dried blood spots and infant stool			[94]
			AAA Cohort (3rd trimester): ↓Nordeoxycholic acid ↑7α-hydroxy-3-oxo-4-cholestenoic acid ↑Stearidonic acid<br><br>Both Cohorts: Pathways related to glycerophospholipid metabolism	MMS Cohort (cumulative pregnancy and 2nd trimester): ↑Glusosamine-6-phosphate	MMS Cohort (cumulative pregnancy): ↓Isoleucine ↓Phenylalanine Metabolism of tyrosine, histidine, tryptophan, and pyrimidine.<br><br>Both Cohorts: Pathways related to methionine and cysteine metabolism, and tryptophan metabolism.	

Table S3. Overview of studies on tobacco smoke- and e-cigarette vapor-associated metabolomic changes in lipid, carbohydrate, and amino acid metabolism related to oxidative stress pathways

Exposure	Population	Metabolomic Method/Metabolite	Lipid Metabolism	Carbohydrate Metabolism	Amino Acid Metabolism	Reference

		Identification				
Maternal smoking was assessed by self-reports via postal questionnaires in early, mid, and late pregnancy. Additionally, environmental tobacco smoke exposure was evaluated based on paternal self-reports.	Mother-infant pairs (N=828) from the Generation R study, Rotterdam, Netherlands, were recruited between April 2002 and January 2006.	Targeted analyses via LC-MS/MS. Authentic standards were used for the identification of amino acids, non-esterified fatty acids (NEFA), phospholipids, and carnitines (Carn). The results were compared to non-exposed neonates.	Neonatal cord blood serum			[103]
			↓Mono-unsaturated acyl-lysophosphatidyl-cholines (Lyso.PC.a), Lyso.PC.e.16:0 and Lyso.PC.e.18:1 concentration, with the strongest relationship for mothers that smoked ≥ 5 cigarettes/day. Sex-specific differences were found, with girls showing ↓Lyso.PC.e concentrations upon continued maternal smoking during pregnancy.	Altered citric acid cycle metabolism in neonates exposed during the first trimester only, and metabolic markers of insulin resistance with continued exposure throughout pregnancy.	Dose/response relationship was strongest for maternal smoking <5 cigarettes/day with Asn/Asp ratio. ↑Neonatal Gln/Glu and Pro/Glu ratio in mothers smoking ≥ 5 cigarettes/day during the first trimester.	
Maternal smoking during the third trimester was evaluated by birth certificate and maternal self-report by questionnaires.	Mother-infant pairs from two cohorts: PRAMS (N=8600, recruited between 2009 and 2019) and INSPIRE (N=1918, recruited between 2012 and 2014), Tennessee, USA.	Targeted analyses via Tandem Mass Spectrometry. Concentration of 33 targeted metabolites, including free carnitine, 21 acylcarnitines, and 11 amino acids, were determined in infants. Only infants with metabolite levels within normal reference ranges were included.	Newborn dried blood spots			[104]
			Higher free carnitine at birth was associated with maternal smoking during the third trimester. Smoking cessation was associated with lower free carnitine compared to continued smoking, with concentrations approaching those observed in infants of	N/A	Higher glycine and leucine at birth were associated with maternal smoking during the third trimester. Smoking cessation was linked to lower levels compared to continued smoking, with concentrations approaching those observed in infants of non-smoking mothers.	

			non-smoking mothers.			
Maternal smoking during pregnancy evaluated by questionnaires and confirmed by urinary cotinine, S-phenyl mercapturic acid and S-benzyl mercapturic acid.	Mother-infant pairs (N=35, 17 smoking and 18 non-smoking mothers) from the LINA study, Leipzig, Germany, were recruited between May 2006 and December 2008. Maternal blood serum samples were collected at the 34th week of gestation and neonatal cord blood samples were collected at birth.	Targeted lipidomics via FIA-MS/MS. Concentration of metabolites using Absolute IDQ p150 Kit, including amino acids, acylcarnitines, hexoses, phosphatidylcholines and sphingomyelines, were determined.	Maternal serum and neonatal cord blood serum			[105]
			Downregulation of PCaa and PCae and SM in maternal serum, but upregulation in cord blood serum of neonates (except for acylcarnitines which were downregulated in newborns of smoking mothers) compared to non-smoking control.	N/A	Upregulation of amino acids, including glutamine, tyrosine, methionine, phenylalanine and glycine in maternal serum, but decreased concentrations in neonatal cord blood serum.	
Low-level nicotine exposure (non-smokers, light smokers or passive exposure to secondhand smoke) was assessed by maternal serum cotinine level (<2–10 ng/mL).	Pregnant women (N=65) who underwent amniocentesis in the second trimester, with fetuses confirmed to have a normal karyotype (2004–2014), from Greenwood, SC, USA. Biobanked samples.	Untargeted LC-HR-MS analysis. Identification was based on accurate m/z, authentic standards, and MS/MS criteria, with error tolerance within 10 ppm and 30 s using xMSanalyzer. Low-level exposure vs. minimal or no exposure groups were compared.	Amniotic fluid			[106]
			Alterations in fatty acid and bile acid metabolism.	Alterations in pyruvate metabolism, TCA cycle, and hexose phosphorylation.	↓Proline<br>↑Arginine<br>Alterations in aspartate and asparagine metabolism, including decreased acetylated polyamines (↓N1,N12-diacetylspermine, N1-acetylspermine, acetylspermidine)	
Maternal exposure to tobacco smoke during pregnancy was evaluated by measuring maternal cotinine	Pregnant women (N=105) from the Atlanta African-American Maternal-Child cohort (2014–2016); samples and	Untargeted LC-HRMS analyses. MWAS and meet-in-the-middle approach. Identification was based on accurate	Non-fasting maternal serum			[107]
			↑LysoPC(16:0)<br>↑LysoPE(18:0)<br>↑LysoPC(18:0)<br>(alterations in fatty acid metabolism),	↑N-acetylneuraminate<br>↑Maltose	↑Tyramine<br>↑Serine<br>↑Glutamate<br>↑Choline<br>↑Taurine	

concentrations in spot urine at two time points during pregnancy. The median urinary cotinine concentration was 5.93 µg/g creatinine in early pregnancy and 3.69 µg/g creatinine in late pregnancy.	data were collected at 8–14 and 24–30 weeks gestation.	mass (m/z), retention time, authentic standards and database matching (METLIN, ChemSpider, HMDB, and KEGG) with a 10 ppm mass error threshold.	↑Ethanolamine phosphate ↓17-hydroxyprogesterone ↑Glycerol-2-phosphate			
Maternal smoking during pregnancy evaluation was based on data from birth certificate and levels of cotinine and hydroxycotinine in neonatal dried blood spots.	Cancer free children (N=899) born between 1983 and 2011 from California, USA.	Untargeted LC-HRM analysis, using MWAS approach for data analysis. Identification of metabolites were confirmed by using authentic standards.	Neonatal dried blood spot			[112]
			↑Retinol (Vit A) ↑FA (Gondoic acid) ↑Arachidic acid	↑Adenosine-5’-diphosphoglucose ↑Gluconic acid	↑Kynurenine ↑Alanine ↑L-Tyrosine ↓Cystine	
Second-hand exposure to e-cigarettes during first trimester (<18 weeks gestation), self-report	Pregnant women (N=73, of whom 55 unexposed and 18 exposed) from New York, USA, in first trimester (April 2017 to January 2019). Ongoing, prospective longitudinal study	Untargeted LC-HRMS, reverse-phase strategy. NIST17MS/MS, and METLIN databases, MS^2 library spectrum match, manual annotation and source inference using PubChem and HMDB. Mass error tolerance: 15 ppm	Maternal urinary metabolome			[115]
			↑2,4-undecadiene-8,10-diynoic acid isobutylamide, ↑palmitamide, ↑glycerol trihexanoate	↓sedoheptulose-7-phosphate (a metabolite in the pentose phosphate pathway)	↑N-acetylputrescine ↓5-methoxytryptophan and ↑5-hydroxy-L-tryptophan	

Abbreviations:

Asn	Asparagine
Asp	Aspartic acid
BEAR Cohort	Biomarkers of Exposure to Arsenic
CALINE4	The California Line Source Dispersion Model, version 4
EWAS	Environment, and Social stressors
FIA-MS/MS	Flow injection analysis coupled with tandem mass spectrometry
GDM	Gestational diabetes mellitus
Gln	Glutamine
Glu	Glutamic acid
HMDB	Human Metabolome Database
iAs	Inorganic arsenic
INSPIRE Cohort	Infant Susceptibility to Pulmonary Infections and Asthma following Respiratory Syncytial Virus (RSV) Exposure
KEGG	Kyoto Encyclopedia of Genes and Genomes
LC-HRMS	Liquid Chromatography – High Resolution Mass Spectrometry
LINA Study	Lifestyle and Environmental Factors and their Influence on Newborn Allergy Risk
LPA	Lysophosphatidic acid
LPC	Lysophosphatidylcholine
LPG	Lysophosphatidylglycerol
Lyso.PC.a	Acyl-lysophosphatidylcholines
Lyso.PC.e	Alkyl-lysophosphatidylcholines
MADRES study	The Maternal and Developmental Risks from Environmental and Social Stressors
MIMA	Meet-in-metabolite-analysis
MITM	Meet-in-the-middle approach
MSE	Mass Spectrometry with Elevated Energy, untargeted fragmentation of all ions using alternating collision energies for wide metabolite coverage
MS/MS	Tandem mass spectrometry with targeted fragmentation of selected molecules to help identify compounds

MWAS	Metabolome-wide association approach
NIST	National Institute of Standards and Technology
PCaa	Diacyl-phosphatidylcholine
PCae	Acyl-alkyl-phosphatidylcholine
PHIME Cohort	Public Health Impact of long-term, low-level Mixed Element exposure in susceptible population strata
PRAMS	Tennessee Pregnancy Risk Assessment Monitoring System
PROGRESS Cohort	Programming Research in Obesity, Growth, Environment and Social Stressors
Pro	Proline
PROTECT Cohort	Puerto Rico Testsite for Exploring Contamination Threats
PTB	Pre-term birth
SM	Sphingomyelins
TAP data	Tracking Air Pollution in China (a combined geographic–statistical model utilizing satellite remote sensing data)
tAs	Total arsenic

References

24. Chen, W.; Qiu, C.; Hao, J.; Liao, J.; Lurmann, F.; Pavlovic, N.; Habre, R.; Jones, D.P.; Bastain, T.M.; Breton, C.V.; et al. Maternal metabolomics linking prenatal exposure to fine particulate matter and birth weight: A cross-sectional analysis of the MADRES cohort. *Environ. Health* **2025**, *24*, 14. https://doi.org/10.1186/s12940-025-01162-x.
58. Li, H.; Huang, K.; Jin, S.; Peng, Y.; Liu, W.; Wang, M.; Zhang, H.; Zhang, B.; Xia, W.; Li, Y.; et al. Environmental cadmium exposure induces alterations in the urinary metabolic profile of pregnant women. *Int. J. Hyg. Environ. Health* **2019**, *222*, 556–562. https://doi.org/10.1016/j.ijheh.2019.02.007.
59. Li, Y.; Zhang, L.; Liu, J.; Wu, M.; Li, C.; Yang, J.; Wang, L. Environmental concentrations of cadmium and zinc and associating metabolomics profile alternations in urine of pregnant women in the first trimester: A prospective cohort study in Taiyuan, North China. *Ecotoxicol. Environ. Saf.* **2023**, *267*, 115611. https://doi.org/10.1016/j.ecoenv.2023.115611.
60. Niedzwiecki, M.M.; Eggers, S.; Joshi, A.; Dolios, G.; Cantoral, A.; Lamadrid-Figueroa, H.; Amarasiriwardena, C.; Téllez-Rojo, M.M.; Wright, R.O.; Petrick, L. Lead exposure and serum metabolite profiles in pregnant women in Mexico City. *Environ. Health* **2021**, *20*, 125. https://doi.org/10.1186/s12940-021-00810-2.
61. Li, H.; Wang, M.; Liang, Q.; Jin, S.; Sun, X.; Jiang, Y.; Pan, X.; Zhou, Y.; Peng, Y.; Zhang, B.; et al. Urinary metabolomics revealed arsenic exposure related to metabolic alterations in general Chinese pregnant women. *J. Chromatogr. A* **2017**, *1479*, 145–152. https://doi.org/10.1016/j.chroma.2016.12.007.
62. Zhang, Q.; Tian, M.; Zhang, X.; Zhang, X.; Yang, X.; Lu, Y.Y.; Li, S.; Liu, L.; Li, J.; Hassanian-Moghaddam, H.; et al. Metabolic biomarkers linking urinary arsenic species to gestational diabetes mellitus: A cross-sectional study in Chinese pregnant women. *Sci. Total Environ.* **2023**, *892*, 164761. https://doi.org/10.1016/j.scitotenv.2023.164761.

65. Laine, J.E.; Bailey, K.A.; Olshan, A.F.; Smeester, L.; Drobná, Z.; Stýblo, M.; Douillet, C.; García-Vargas, G.; Rubio-Andrade, M.; Pathmasiri, W.; et al. Neonatal metabolomic profiles related to prenatal arsenic exposure. *Environ. Sci. Technol*. **2017**, *51*, 625–633. https://doi.org/10.1021/acs.est.6b04374.
68. Zhang, M.; Buckley, J.P.; Liang, L.; Hong, X.; Wang, G.; Wang, M.C.; Wills-Karp, M.; Wang, X.; Mueller, N.T. A metabolome-wide association study of in utero metal and trace element exposures with cord blood metabolome profile: Findings from the Boston Birth Cohort. *Environ. Int*. **2022**, *158*, 106976. https://doi.org/10.1016/j.envint.2021.106976.
72. Kim, C.; Ashrap, P.; Watkins, D.J.; Mukherjee, B.; Rosario-Pabón, Z.Y.; Vélez-Vega, C.M.; Alshawabkeh, A.N.; Cordero, J.F.; Meeker, J.D. Maternal metals/metalloid blood levels are associated with lipidomic profiles among pregnant women in Puerto Rico. *Front. Public Health* **2022**, *9*, 754706. https://doi.org/10.3389/fpubh.2021.754706.
73. Zhao, S.; Yang, X.; Xu, Q.; Li, H.; Su, Y.; Xu, Q.; Li, Q.; Xia, Y.; Shen, R. Association of maternal metals exposure, metabolites and birth outcomes in newborns: A prospective cohort study. *Environ. Int*. **2023**, *179*, 108183. https://doi.org/10.1016/j.envint.2023.108183.
74. Xie, Y.; Xiao, H.; Zheng, D.; Mahai, G.; Li, Y.; Xia, W.; Xu, S.; Zhou, A. Associations of prenatal metal exposure with child neurodevelopment and mediation by perturbation of metabolic pathways. *Nat. Commun.* **2025**, *16*, 2089. https://doi.org/10.1038/s41467-025-57253-3.
75. Anesti, O.; Papaioannou, N.; Gabriel, C.; Karakoltzidis, A.; Dzhedzheia, V.; Petridis, I.; Stratidakis, A.; Dickinson, M.; Horvat, M.; Snoj Tratnik, J.; et al. An exposome connectivity paradigm for the mechanistic assessment of the effects of prenatal and early life exposure to metals on neurodevelopment. *Front. Public Health* **2023**, *10*, 871218. https://doi.org/10.3389/fpubh.2022.871218.
76. Wang, M.; Xia, W.; Liu, H.; Liu, F.; Li, H.; Chang, H.; Sun, J.; Liu, W.; Sun, X.; Jiang, Y.; et al. Urinary metabolomics reveals novel interactions between metal exposure and amino acid metabolic stress during pregnancy. *Toxicol. Res.* **2018**, *7*, 1164–1172. https://doi.org/10.1039/c8tx00042e.
77. Wu, H.; Xu, B.; Guan, Y.; Chen, T.; Huang, R.; Zhang, T.; Sun, R.; Xie, K.; Chen, M. A metabolomic study on the association of exposure to heavy metals in the first trimester with primary tooth eruption. *Sci. Total Environ.* **2020**, *723*, 138107. https://doi.org/10.1016/j.scitotenv.2020.138107.
81. Zheng, L.; Zhou, J.; Zhu, L.; Xu, X.; Luo, S.; Xie, X.; Li, H.; Lin, S.; Luo, J.; Wu, S. Associations of air pollutants and related metabolites with preterm birth during pregnancy. *Sci. Total Environ*. **2024**, *951*, 175542. https://doi.org/10.1016/j.scitotenv.2024.175542.
82. Li, Z.; Dunlop, A.L.; Sarnat, J.A.; Hüls, A.; Eick, S.M.; Gaskins, A.; Chang, H.; Russell, A.; Tan, Y.; Cheng, H.; et al. Unraveling the molecular links between fine particulate matter exposure and early birth risks in African American mothers: A metabolomics study in the Atlanta African American Maternal-Child Cohort. *Environ. Sci. Technol*. **2025**, *59*, 10905–10918. https://doi.org/10.1021/acs.est.5c02071.
84. Wang, K.; Zhang, L.; Li, Q.; Xu, S.; Wang, P.; Shi, H.; Zhang, Y.; Li, J. The effect of PM2.5 exposure on placenta and its associated metabolites: A birth cohort study. *Ecotoxicol. Environ. Saf.* **2025**, *292*, 117891. https://doi.org/10.1016/j.ecoenv.2025.117891.
85. Yan, Q.; Liew, Z.; Uppal, K.; Cui, X.; Ling, C.; Heck, J.E.; von Ehrenstein, O.S.; Wu, J.; Walker, D.I.; Jones, D.P.; et al. Maternal serum metabolome and traffic-related air pollution exposure in pregnancy. *Environ. Int*. **2019**, *130*, 104872. https://doi.org/10.1016/j.envint.2019.05.066.
86. Kim, J.H.; Yan, Q.; Uppal, K.; Cui, X.; Ling, C.; Walker, D.I.; Heck, J.E.; von Ehrenstein, O.S.; Jones, D.P.; Ritz, B. Metabolomics analysis of maternal serum exposed to high air pollution during pregnancy and risk of autism spectrum disorder in offspring. *Environ. Res*. **2021**, *196*, 110823. https://doi.org/10.1016/j.envres.2021.110823.

89. India-Aldana, S.; Petrick, L.; Niedzwiecki, M.M.; Valvi, D.; Just, A.C.; Gutiérrez-Avila, I.; Kloog, I.; Barupal, D.K.; Téllez-Rojo, M.M.; Wright, R.O.; et al. Pregnancy as a susceptible period to ambient air pollution exposure on the maternal postpartum metabolome. *Environ. Sci. Technol*. **2025**, *59*, 6400–6413. https://doi.org/10.1021/acs.est.4c10717.
90. Zhang, J.; Chen, G.; Liang, S.; Liu, J.; Zhang, J.; Shen, H.; Chen, Y.; Duan, J.; Sun, Z. PM2.5 exposure exaggerates the risk of adverse birth outcomes in pregnant women with pre-existing hyperlipidemia: Modulation role of adipokines and lipidome. *Sci. Total Environ.* **2021**, *787*, 147604. https://doi.org/10.1016/j.scitotenv.2021.147604.
91. Ritz, B.; Yan, Q.; He, D.; Wu, J.; Walker, D.I.; Uppal, K.; Jones, D.P.; Heck, J.E. Child serum metabolome and traffic-related air pollution exposure in pregnancy. *Environ. Res.* **2022**, *203*, 111907. https://doi.org/10.1016/j.envres.2021.111907.
93. Holzhausen, E.A.; Chalifour, B.N.; Tan, Y.; Young, N.; Lurmann, F.; Jones, D.P.; Sarnat, J.A.; Chang, H.H.; Goran, M.I.; Liang, D.; et al. Prenatal and early life exposure to ambient air pollutants is associated with the fecal metabolome in the first two years of life. *Environ. Sci. Technol.* **2024**, *58*, 14121–14134. https://doi.org/10.1021/acs.est.4c02929.
94. Holzhausen, E.A.; Tan, Y.; Young, N.; Jones, R.B.; Tang, Z.; Sarnat, J.A.; Lurmann, F.; Chang, H.H.; Tran, V.; Jones, D.P.; et al. Prenatal nitrogen oxide (NOx) and its potential impact on infant metabolism during the first month of life: Evidence from two distinct cohorts—The Atlanta African American Maternal-Child Cohort and the Southern California Mother's Milk Study. *Environ. Sci. Technol.* **2025**, *59*, 19131–19145. https://doi.org/10.1021/acs.est.5c04955.
103. Cajachagua-Torres, K.N.; Blaauwendraad, S.M.; El Marroun, H.; Demmelmair, H.; Koletzko, B.; Gaillard, R.; Jaddoe, V.W.V. Fetal exposure to maternal smoking and neonatal metabolite profiles. *Metabolites* **2022**, *12*, 1101. https://doi.org/10.3390/metabo12111101.
104. Snyder, B.M.; Nian, H.; Miller, A.M.; Ryckman, K.K.; Li, Y.; Tindle, H.A.; Ammar, L.; Ramesh, A.; Liu, Z.; Hartert, T.V.; et al. Associations between smoking and smoking cessation during pregnancy and newborn metabolite concentrations: Findings from PRAMS and INSPIRE birth cohorts. *Metabolites* **2023**, *13*, 1163. https://doi.org/10.3390/metabo13111163.
105. Rolle-Kampczyk, U.E.; Krumsiek, J.; Otto, W.; Röder, S.W.; Kohajda, T.; Borte, M.; Theis, F.; Lehmann, I.; von Bergen, M. Metabolomics reveals effects of maternal smoking on endogenous metabolites from lipid metabolism in cord blood of newborns. *Metabolomics* **2016**, *12*, 76. https://doi.org/10.1007/s11306-016-0983-z.
106. Fischer, S.T.; Lili, L.N.; Li, S.; Tran, V.T.; Stewart, K.B.; Schwartz, C.E.; Jones, D.P.; Sherman, S.L.; Fridovich-Keil, J.L. Low-level maternal exposure to nicotine associates with significant metabolic perturbations in second-trimester amniotic fluid. *Environ. Int.* **2017**, *107*, 227–234. https://doi.org/10.1016/j.envint.2017.07.019.
107. Tan, Y.; Barr, D.B.; Ryan, P.B.; Fedirko, V.; Sarnat, J.A.; Gaskins, A.J.; Chang, C.J.; Tang, Z.; Marsit, C.J.; Corwin, E.J.; et al. High-resolution metabolomics of exposure to tobacco smoke during pregnancy and adverse birth outcomes in the Atlanta African American maternal-child cohort. *Environ. Pollut.* **2022**, *292 Pt A*, 118361. https://doi.org/10.1016/j.envpol.2021.118361.
112. He, D.; Yan, Q.; Uppal, K.; Walker, D.I.; Jones, D.P.; Ritz, B.; Heck, J.E. An untargeted metabolome-wide association study of maternal perinatal tobacco smoking in newborn blood spots. *Metabolomics* **2025**, *21*, 30. https://doi.org/10.1007/s11306-025-02225-3.
115. Cavalier, H.; Long, S.E.; Rodrick, T.; Siu, Y.; Jacobson, M.H.; Afanasyeva, Y.; Sherman, S.; Liu, M.; Kahn, L.G.; Jones, D.R.; et al. Exploratory Untargeted Metabolomics Analysis Reveals Differences in Metabolite Profiles in Pregnant People Exposed vs. Unexposed to E-Cigarettes Secondhand in the NYU Children's Health and Environment Study. *Metabolomics* **2025**, *21*, 92. https://doi.org/10.1007/s11306-025-02280-w.
